# The Antipsychotic Drug Aripiprazole Suppresses Colorectal Cancer by Targeting LAMP2a to Induce RNH1/miR‐99a/mTOR‐Mediated Autophagy and Apoptosis

**DOI:** 10.1002/advs.202409498

**Published:** 2024-11-08

**Authors:** Hui‐Fang Hu, Jia‐Ying Fu, Lei Han, Gui‐Bin Gao, Wei‐Xia Zhang, Si‐Ming Yu, Nan Li, Yang‐Jia Li, Yi‐Fan Lu, Xiao‐Feng Ding, Yun‐Long Pan, Yang Wang, Qing‐Yu He

**Affiliations:** ^1^ MOE Key Laboratory of Tumor Molecular Biology and State Key Laboratory of Bioactive Molecules and Druggability Assessment College of Life Science and Technology Jinan University Guangzhou 510632 China; ^2^ The First Affiliated Hospital of Jinan University and MOE Key Laboratory of Tumor Molecular Biology Jinan University Guangzhou 510632 China; ^3^ Institute of Biomedicine and National Engineering Research Center of Genetic Medicine and Key Laboratory of Biomaterials of Guangdong Higher Education Institutes National Engineering Research Centre of Genetic Medicine College of Life Science and Technology Jinan University Guangzhou 510632 China

**Keywords:** 5‐FU, autophagy, aripiprazole, LAMP2a, mTOR

## Abstract

The mammalian target of rapamycin (mTOR) is a critical signaling hub for sustaining cancer survival. Targeting mTOR and inducing autophagic cell death downstream of it represent promising therapeutic strategies for cancer prevention. A US Food and Drug Administration‐approved drug library containing 616 small molecules is used to screen anticancer drugs against colorectal cancer (CRC) cells that rely on mTOR. This led to the identification of an antipsychotic drug aripiprazole, which significantly induced mTOR inhibition and autophagic apoptosis in CRC, in vitro and in vivo. The use of drug affinity response target stability identified lysosome‐associated membrane protein 2A (LAMP2a) as a direct target of aripiprazole. LAMP2a‐deficient CRC cells are refractory to aripiprazole. High LAMP2a expression is associated with poor survival of patients with CRC and negatively correlated with expression of ribonuclease inhibitor 1 (RNH1), which is later confirmed as a novel substrate of LAMP2a. Mechanistically, aripiprazole bound to the Lys401–His404 of LAMP2a and repressed its activity, subsequently inactivating RNH1/miR‐99a/mTOR signaling and inducing autophagy‐mediated apoptosis, thereby suppressing tumorigenesis. Liposome‐mediated delivery of aripiprazole in combination with fluorouracil elicited superior therapeutic benefits in CRC, as compared to single treatments, thereby highlighting that aripiprazole may be repurposed as a novel therapeutic agent for CRC treatment.

## Graphical abstract

Aripiprazole directly interacts with LAMP2a to suppress CRC tumorigenesis. By coordinating with LAMP2a, aripiprazole regulates the ribonuclease inhibitor 1 (RNH1)/microRNA (miR)‐99a/mTOR signaling pathway, to induce autophagy and subsequent apoptosis, thereby improving the antitumor effects of fluorouracil (5‐FU). The data indicate that aripiprazole can be repurposed as an anticancer drug for adjuvant therapy of CRC. Its binding to LAMP2a reveals a hidden link between CMA and macroautophagy.

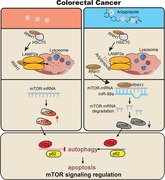

## 1 Introduction

Colorectal cancer (CRC) is one of the most common malignant tumors with poor overall survival, having the incidence and mortality respectively ranking 3rd and 2nd worldwide.^[^ ^1^ ^]^ Failure of radiotherapy and chemotherapy is the main cause of poor prognosis in cancer patients, and radiation and chemical agents damage both cancer and normal cells, leading to serious adverse effects. Therefore, there is an urgent need to identify new biomarkers and explore effective and safe agents for CRC therapy.

The mammalian target of rapamycin (mTOR) is a master regulator of cellular metabolism and is highly activated in most human cancers.^[^ ^2^ ^]^ The activation of mTOR leads to the inhibition of autophagy through the phosphorylation of multiple autophagy‐related proteins, such as serine/threonine‐protein kinase ULK1 (ULK1), autophagy‐related protein 13 (ATG13), and beclin 1‐associated autophagy‐related key regulator (ATG14L), which promote autophagy initiation and autophagosome nucleation.^[^ ^2^, ^3^, ^4^ ^]^ Many anticancer drugs have been reported to exert their anticancer activity by inducing autophagy.^[^ ^5^ ^]^ Therefore, targeting autophagy and mTOR signaling may offer a therapeutic strategy to inhibit tumor progression. Most available mTOR inhibitors that have been rigorously tested for clinical use are rapamycin derivatives; however, the performance of rapamycin and its analogs has been undistinguished despite isolated successes in subsets of cancer,^[^ ^6^ ^]^ suggesting that the full therapeutic potential of targeting mTOR is yet to be exploited.

Drug repurposing is a promising strategy for drug development owing to the well‐recognized characterization of the physicochemistry, pharmacokinetics, and safety of known drugs.^[^ ^7^ ^]^ Its advantages include a high efficiency, short duration of development, and low cost. Successful cases of drug repurposing have been reported, including warfarin and leflunomide.^[^ ^7^, ^8^ ^]^ US Food and Drug Administration (FDA)‐approved drugs are an excellent resource for drug repurposing to screen novel cancer therapeutics. By means of high‐throughput screening of an FDA‐approved drug library, aripiprazole, an atypical antipsychotic drug that has been reported to be a partial agonist of high‐affinity 5‐hydroxytryptamine receptor 1A (HTR1A),^[^ ^9^ ^]^ was found to significantly suppress the growth of HCT116 cells (high mTOR expression), but not that of HT29 cells (low mTOR expression). However, the mechanism through which aripiprazole regulates mTOR expression in CRC remains unclear.

Drug affinity response target stability (DARTS) has been successfully employed as an efficient method to identify target proteins of compounds.^[^ ^10^ ^]^ Using DARTS combined with mass spectrometry (MS)‐based proteomics, we identified lysosome‐associated membrane protein 2A (LAMP2a) as a direct target of aripiprazole. LAMP2a plays an important role in chaperone‐mediated autophagy (CMA),^[^ ^11^ ^]^ a biological process involving the lysosomal degradation of proteins carrying a recognizable peptide sequence motif, KFERQ.^[^ ^12^, ^13^ ^]^ The molecular chaperone heat shock cognate 71 kDa protein (HSC70) recognizes proteins bearing the motif and translocates them to lysosomes for degradation, via binding to LAMP2a.^[^ ^14^ ^]^ CMA and LAMP2a are highly activated in various malignant tumors.^[^ ^13^, ^15^, ^16^ ^]^

In this study, the antitumor properties and mechanism of aripiprazole were investigated, using a series of in vitro and in vivo experiments. We found that, in addition to working on its original antipsychotic target, HTR1A, aripiprazole directly interacts with LAMP2a to suppress CRC tumorigenesis. By coordinating with LAMP2a, aripiprazole regulates the ribonuclease inhibitor 1 (RNH1)/microRNA (miR)‐99a/mTOR signaling pathway, to induce autophagy and subsequent apoptosis, thereby improving the antitumor effects of fluorouracil (5‐FU). Our data indicate that aripiprazole can be repurposed as an anticancer drug for adjuvant therapy of CRC. Its binding to LAMP2a reveals a hidden link between CMA and macroautophagy.

## 2 Results

### 2.1 Aripiprazole Suppresses CRC Cells with High Level of mTOR

**Figure 1:**
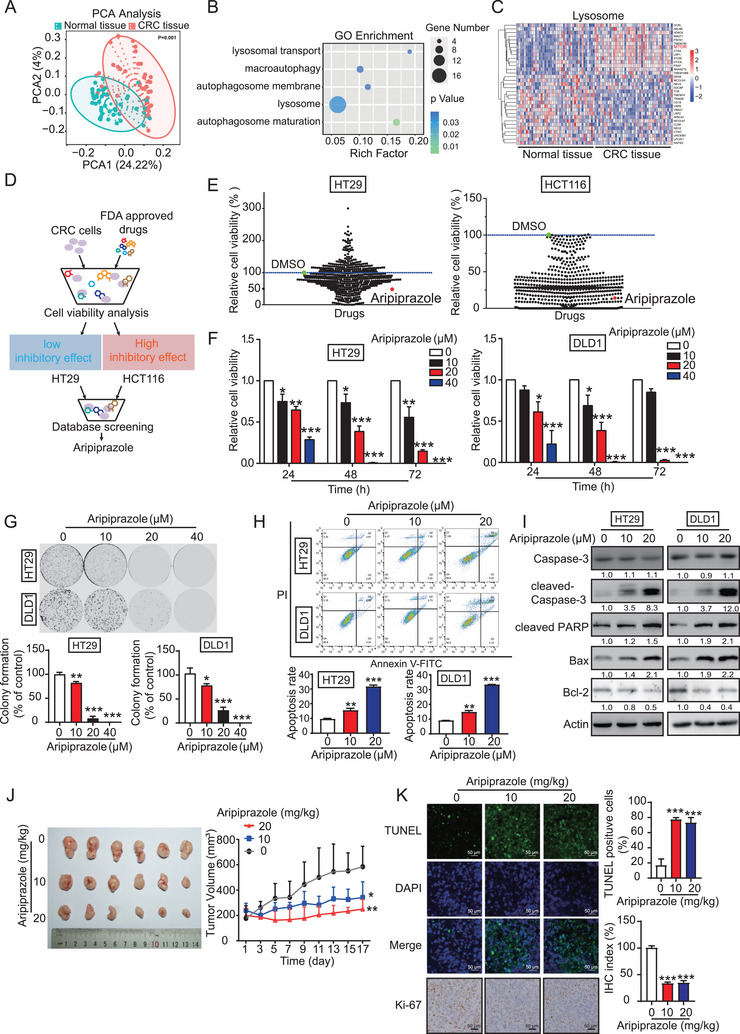
Aripiprazole suppresses the tumorigenesis of CRC cells with high level of mTOR. A) Principal component analysis of GEO dataset (GSE115313), which includes 42 CRC and paired normal tissues. B) Gene Ontology pathway analysis of the DEPs between CRC and paired normal tissues. C) Hierarchical clustering analysis showing the DEPs were involved in the lysosome pathway. D) Schematic diagram for identifying anticancer drugs amongst an US Food and Drug Administration‐approved small‐molecule library (616 compounds) using WST‐1 assay. E) HT29 cells were treated with the 616 drugs (10 µm) individually or with dimethyl sulfoxide for 72 h, followed by WST‐1 assay. F,G) HT29 and DLD1 cells were treated with increasing concentrations of aripiprazole (10–40 µm), the cell viability and colony‐formation ability were analyzed using WST‐1 assay (F) and colony‐formation assay (G), respectively, *n* = 3/experiments. H,I) Comparison of cell apoptosis and relative apoptotic markers in CRC cells treated with various concentrations of aripiprazole (10–20 µm, 48 h) using Annexin V‐fluorescein isothiocyanate/propidium iodide double staining (H) and western blot (I), *n* = 3/experiments. J) Mice bearing HT29‐derived tumor xenografts were intragastrically administered with aripiprazole (10 or 20 mg kg^−1^) once every 2 d, *n* = 6 mice/group. K) Cell apoptosis and the tumor growth index in tumor xenografts were analyzed by TUNEL and Ki‐67 staining assays, respectively, *n* = 3 mice/group. Bars, SD; ^*^ *p* < 0.05; ^**^ *p* < 0.01; ^***^ *p* < 0.001; ns, no significant difference.

To investigate the key signaling pathways and biological processes involved in CRC progression, we analyzed the National Center for Biotechnology Information Gene Expression Omnibus (GEO) dataset GSE115313, which included 42 CRC and paired normal tissues (**Figure** **1**A). Gene Ontology analysis revealed that lysosomes and autophagy are key pathways in CRC (Figure 1B). Moreover, hierarchical clustering analysis identified two clusters, characterized by strong and weak expression of the lysosome signature. We found that mTOR was significantly upregulated in CRC tissues (Figure 1C), as well as in malignant CRC cell lines, but not in normal colonic epithelial cells (NCM460; Figure S1A, Supporting Information). These findings inspired us to screen for new anticancer drugs targeting mTOR, to suppress CRC. Based on a library comprising of 616 FDA‐approved drugs (Table S2, Supporting Information), two CRC cell lines, HCT116 (high level of mTOR) and HT29 (low level of mTOR), were subjected to cell viability analysis (10 µM, 72 h; Figure 1D,E). Aripiprazole, an atypical antipsychotic drug, was found to exert a significant inhibitory effect on HCT116 cells, but a moderate effect on HT29 cells; this became our focus going forward. We then determined the sensitivity of several CRC cells with different levels of mTOR to aripiprazole (Figure S1A, Supporting Information) and found that aripiprazole had weaker effects on the inhibition of cell proliferation and the induction of apoptosis in RKO and NCM460 cells, which have lower mTOR expression (Figure S1B–D, Supporting Information). On the other hand, in HT29 and DLD1 cells, the high mTOR‐expressing cell lines, aripiprazole significantly inhibited cell viability (Figure 1F) and colony formation (Figure 1G) in a dose‐dependent manner. Next, we performed an Annexin V‐fluorescein isothiocyanate (FITC)/propidium iodide (PI) staining assay to determine the effect of aripiprazole on apoptosis in CRC cells. As shown in Figure 1H, aripiprazole elicited CRC cell apoptosis in a dose‐dependent manner (0–20 µM), which was further confirmed by the increased expressions of cleaved‐Caspase 3, cleaved‐PARP, Bax, and the decreased expression of Bcl2 upon aripiprazole treatment (Figure 1I).

To determine the therapeutic potential of aripiprazole in vivo, HT29 cells were subcutaneously injected into the flanks of nude mice that were orally administered aripiprazole or the vehicle control. As shown in Figure 1J, tumor tissues were harvested after the sacrifice of mice on day 17 for tumor volume comparison, aripiprazole treatment (10 and 20 mg kg^−1^) markedly inhibited the tumor volume by 47.5% and 57.1%, respectively. Ki‐67 staining and TdT‐mediated dUTP nick‐end labeling (TUNEL) assays suggested that aripiprazole significantly inhibited tumor cell proliferation and induced apoptosis, respectively (Figure 1K). No significant differences in body weight were observed between the treatment and control groups (Figure S2A, Supporting Information). Meanwhile, histological examination assay revealed that aripiprazole treatment did not exert any change in terms of histological morphology of the liver, kidney, and lung (Figure S2B, Supporting Information). Furthermore, there were no significant differences in the serum alanine and aspartate aminotransferase levels of nude mice of the treatment and control groups (Figure S2C, Supporting Information). Collectively, our data indicated that aripiprazole has potent antitumor activity against CRC, with no significant side effects.

### 2.2 Aripiprazole Induces Autophagy to Promote Apoptosis in CRC Cells

**Figure 2:**
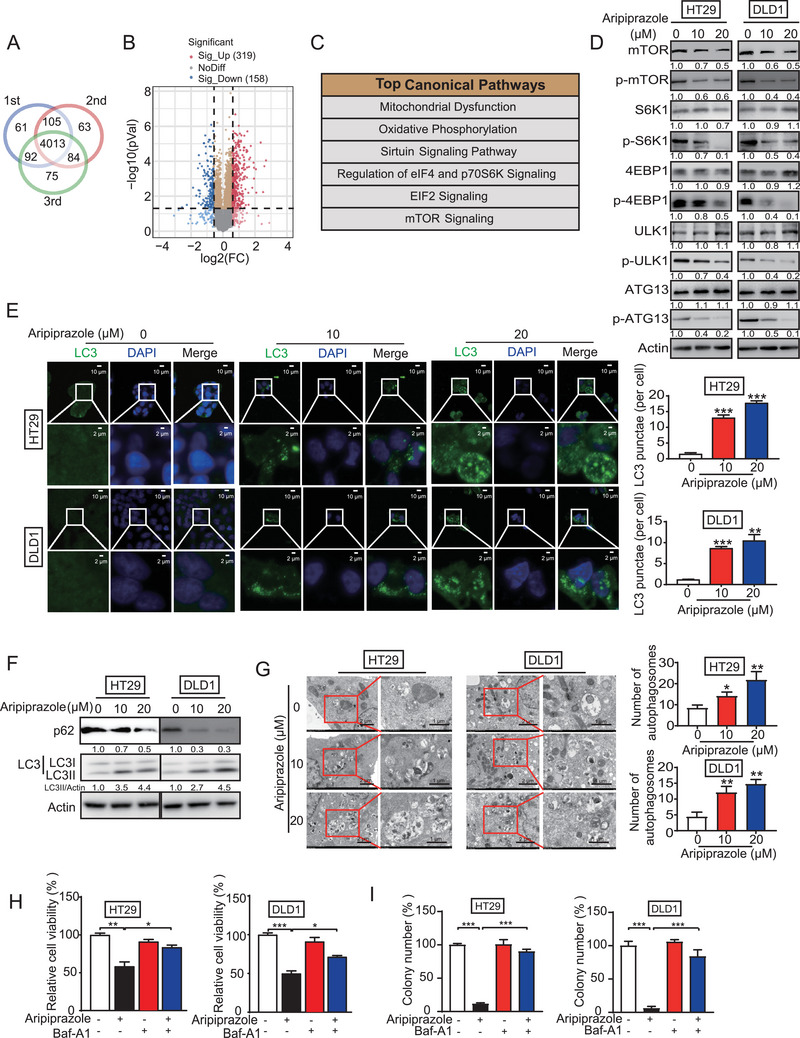
Aripiprazole induces autophagy and apoptosis in CRC cells. A) Overlapping proteins identified in triplicate. B) Volcano plot showing the DEPs regulated by aripiprazole. A total of 477 DEPs (fold‐change ≥1.5, *p‐*value ≤0.05) were identified, including 158 downregulated and 319 upregulated proteins. C) Aripiprazole‐regulated DEPs were analyzed using Ingenuity Pathway Analysis, and the top canonical pathways are shown. D) HT29 and DLD1 cells were exposed to different concentrations of aripiprazole (0–20 µm) for 48 h, and western blot analysis was performed to detect the expression levels of p‐mTOR, p‐S6K1, p‐4EBP1, p‐ULK1, and p‐ATG13, *n* = 3/experiments. E) Immunofluorescence of LC3 in HT29 and DLD1 cells after aripiprazole treatment (0–20 µm) for 48 h. The LC3 puncta in the cells were quantified, *n* = 3/experiments. F) The expression of LC3 and p62 in CRC cells with indicated treatment were analyzed using western blot, *n* = 3/experiments. G) Transmission electron microscopy of HT29 and DLD1 cells treated with aripiprazole (10 and 20 µm) or dimethyl sulfoxide for 48 h, *n* = 3/experiments. H,I) HT29 and DLD1 cells were treated with aripiprazole (20 µm, 48 h) or dimethyl sulfoxide, with or without pretreatment with Baf‐A1 (10 nm, 12 h), following which the cell viability and colony‐formation ability were determined using WST‐1 (H) and colony‐formation (I) assays, respectively, *n* = 3/experiments. Bars, SD; ^*^ *p* < 0.05; ^**^ *p* < 0.01; ^***^ *p* < 0.001; ns, no significant difference.

To investigate the molecular mechanism of aripiprazole in CRC, data‐independent acquisition‐based proteomics was performed to identify the differentially expressed proteins in HT29 cells treated with aripiprazole. As shown in **Figure** **2**A, 4013 proteins were identified in triplicate. The power law global error model was used to determine the protein abundance, with a slope of 0.741 and an adjusted r^2^ of 0.99 (Pearson *r* = 0.747; Figure S3A, Supporting Information, upper panel). Moreover, the data had a fitted normal distribution of the residual standard deviations between the modeled and actual values. The residuals were distributed evenly and independent of the mean abundance rank (Figure S3A, Supporting Information, lower panel). Among the identified proteins, 477 proteins were significantly regulated by aripiprazole, including 319 upregulated and 158 downregulated proteins (fold‐change ≥ 1.5, *p*‐value ≤ 0.05; Figure 2B; Table S3, Supporting Information). Consistently, Ingenuity Pathway Analysis showed that the mTOR signaling pathway plays an important role in the anticancer bioactivity of aripiprazole (Figure 2C). Western blot confirmed that aripiprazole decreased the expressions of mTOR, phospho (p)‐mTOR, and its downstream targets p‐S6K1, p‐4E‐binding protein 1 (4EBP1), p‐ULK1 and p‐ATG13, while having no discernible impact on the total protein expressions of S6K1, 4EBP1, ULK1 and ATG13 (Figure 2D). Notably, aripiprazole also decreased mTOR mRNA levels (Figure S3B, Supporting Information).

Since the mTOR pathway has been reported to inhibit autophagy,^[^ ^5^ ^]^ and our data also indicated that aripiprazole‐regulated proteins are involved in the lysosomal pathway (Figures S3C,D, Supporting Information), we next investigated whether aripiprazole induces autophagy in CRC cells. Confocal microscopy showed that upon aripiprazole treatment, cellular LC3 dots accumulated and co‐localized with the lysosomal marker LAMP1 in HT29 and DLD1 cells (Figure 2E; Figure S3E, Supporting Information). We found that both CRC cell lines showed an increase in LC3II and a decrease in p62 levels after aripiprazole treatment (Figure 2F). Transmission electron microscopy (TEM) analysis showed that upon aripiprazole treatment, HT29 and DLD1 cells developed cellular autophagosome accumulation, in a dose‐dependent manner (Figure 2G). We also found that pretreatment with bafilomycin A1 (Baf‐A1) rescued cell viability (Figure 2H), colony‐formation ability (Figures 2I; Figure S4A, Supporting Information), and apoptosis (Figure S4B, Supporting Information) in aripiprazole‐treated CRC cells. This was also supported by the fact that aripiprazole‐induced cleaved Caspase‐3 was significantly decreased upon pretreatment with Baf‐A1 (Figure S4C, Supporting Information). Moreover, pretreatment with Z‐VAD‐FMK restored the viability of aripiprazole‐treated CRC cells (Figure S4D, Supporting Information), but did not significantly alter aripiprazole‐induced autophagy (indicated by increased LC3II and decreased p62; Figure S4E, Supporting Information), suggesting that aripiprazole‐induced autophagy is upstream of apoptosis. Regarding the important role ATG5 in autophagy process, we employed sh‐RNA against ATG5 and found that knockdown of ATG5 markedly weakened the promotive effect of aripiprazole on CRC cell apoptosis (Figure S4F,G, Supporting Information), suggesting that autophagy occurs before aripiprazole‐mediated apoptosis. Collectively, aripiprazole induces autophagy‐mediated apoptosis by decreasing mTOR expression.

### 2.3 LAMP2a is Essential for the Bioactivity of Aripiprazole

We further investigated the mechanism of how aripiprazole suppresses mTOR. Since HTR1A is the known target of aripiprazole in its original indication,^[^ ^9^ ^]^ we wondered whether aripiprazole exerts its anticancer effect through HTR1A. To address this issue, we established HTR1A‐deficient CRC cells and control cells using CRISPR/Cas9 (Figure S5A, Supporting Information) and found that HTR1A knockout did not abolish the inhibitory effect of aripiprazole on HT29 cells (Figure S5B, Supporting Information), suggesting that in addition to HTR1A, there may be other targets involved in the anticancer function of aripiprazole.

**Figure 3:**
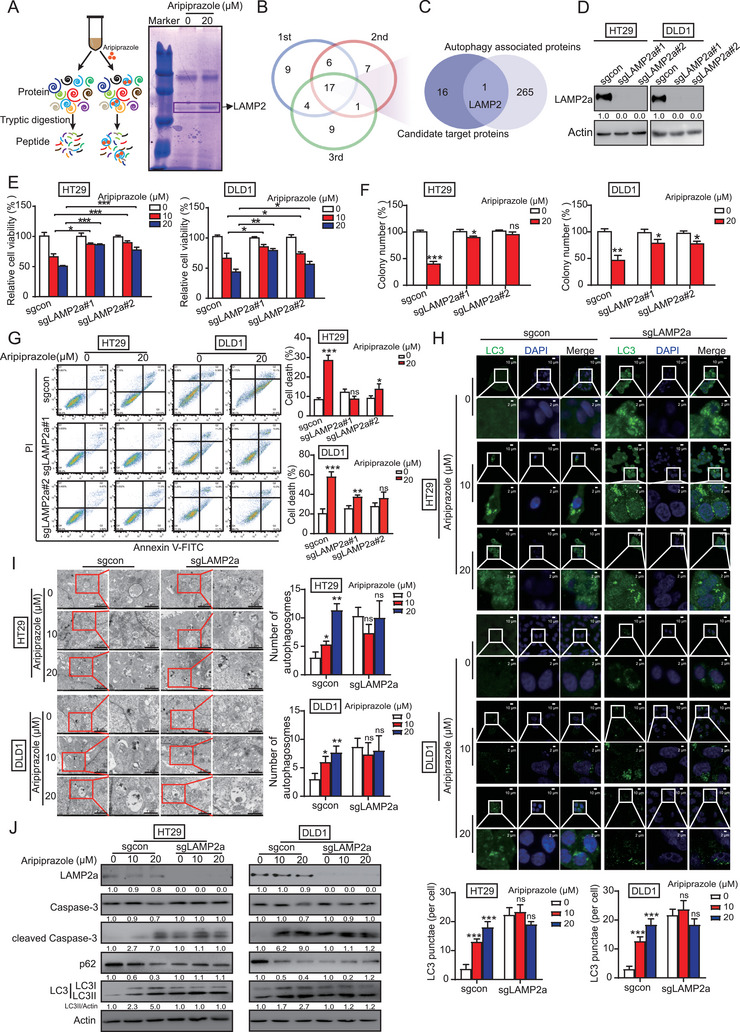
LAMP2a mediates the anticancer effect of aripiprazole in CRC cells. A) Schematic diagram of DARTS technology for identification of aripiprazole targets. Coomassie brilliant blue staining showing the specific band in the aripiprazole treatment lane used for mass spectrometry analysis. B) The target proteins of aripiprazole identified in triplicate were overlapped. C) Venn diagram showing the overlapped proteins between autophagy‐related proteins and DARTS‐identified proteins. D) LAMP2a‐deficient CRC cells were successfully established by CRISPR/Cas9, *n* = 3/experiments. E–G) CRC cells with LAMP2a‐deficiency were treated with aripiprazole (10 and 20 µm) or dimethyl sulfoxide for 48 h, and the viability, colony‐formation ability, and cell apoptosis were determined using WST‐1 (E), colony‐formation (F), and annexin V‐fluorescein isothiocyanate/propidium iodide staining (G) assays, *n* = 3/experiments. H–J) CRC cells with or without LAMP2a‐deficiency were treated with aripiprazole (10 and 20 µm) or dimethyl sulfoxide for 48 h, following which the cellular LC3 puncta were analyzed by immunofluorescence (H) and transmission electron microscopy (I), the expression of apoptotic and autophagic markers in CRC cells were analyzed using western blot (J), *n* = 3/experiments. Bars, SD; ^*^ *p* < 0.05; ^**^ *p* < 0.01; ^***^ *p* < 0.001; ns, no significant difference.

We thus performed DARTS‐MS to identify the target proteins of aripiprazole. As shown in **Figure** **3**A, a specific protein band observed in the aripiprazole‐treated group (Figure 3B) was subjected to MS analysis. We focused on the proteins involved in the autophagy‐lysosome pathway, and thus, lysosome‐associated membrane protein 2 (LAMP2) was selected as a candidate for validation (Figure 3C). We established LAMP2‐deficient CRC cells (Figure S5C, Supporting Information) and found that the anticancer effects of aripiprazole were significantly attenuated in both HT29 and DLD1 cells with LAMP2 knockout (Figure S5D, Supporting Information).

The *LAMP2* gene encodes three alternatively spliced RNA isoforms, *LAMP2A*, *LAMP2B*, and *LAMP2C*, which are distinguishable by a variable transmembrane domain with a 13‐amino acid cytoplasmic tail.^[^ ^17^ ^]^ We subsequently generated LAMP2a‐, LAMP2b‐, and LAMP2c‐deficient CRC cells (Figure S5E,F, Supporting Information), and found that aripiprazole significantly inhibited the viability of cells with LAMP2b or LAMP2c deficiency (Figure S5G, Supporting Information), but not LAMP2a deficiency (Figure 3D–F; Figure S5G,H, Supporting Information). Moreover, knockout of LAMP2a showed comparable inhibitory effect on CRC cell growth with aripiprazole treatment (20 µM, 48 h; Figure S5I, Supporting Information), suggesting that LAMP2a is necessary for the anticancer property of aripiprazole. To further study whether LAMP2a plays a key role in the anticancer effect of aripiprazole, Annexin V‐FITC/PI staining, confocal microscopy, and TEM were performed. The results revealed that aripiprazole‐induced apoptosis and autophagy in HT29 and DLD1 cells were restored by knockout of LAMP2a (Figure 3G–I), which was confirmed by western blot analysis of cleaved Caspase‐3, LC3, and p62 expression (Figure 3J). Taken together, LAMP2a seems to be a target protein of aripiprazole that is essential for its anticancer activity.

### 2.4 Aripiprazole Exerts Its Anticancer Activity by Binding to the Lys401‐His404 of LAMP2a

**Figure 4:**
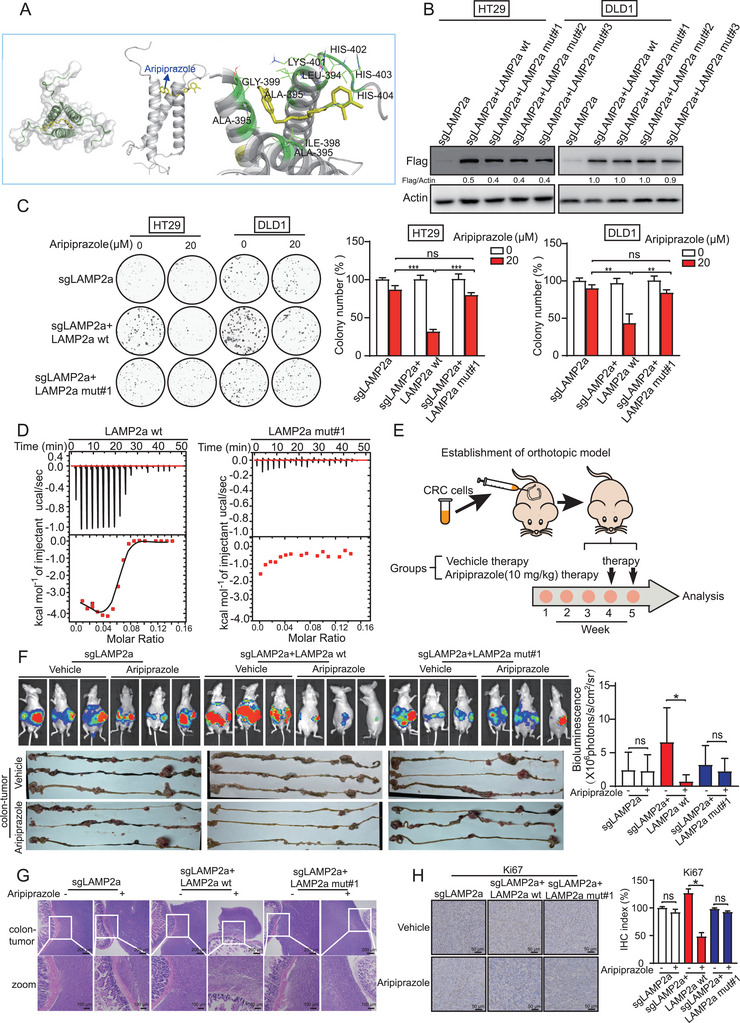
Aripiprazole exhibits its anticancer activity by binding to LAMP2a at Lys401‐His404. A) Molecular docking for prediction of the binding sites of aripiprazole to LAMP2a protein. The potential binding sites are labeled. Yellow, aripiprazole; gray, LAMP2a (PDB: 2MOM). B,C) Wild‐type or mutated LAMP2a were re‐overexpressed in LAMP2a‐deficient HT29 and DLD1 cells, and colony‐formation assay was performed to compare their sensitivity toward aripiprazole treatment (20 µm, 48 h) (C), *n* = 3/experiments. D) Isothermal titration calorimetry assay was performed to determine the interaction between aripiprazole and LAMP2a‐wt or LAMP2a mut#1, *n* = 3/experiments. E) DLD1‐Luc cells were injected to the cecum to establish orthotopic xenograft models in nude mice, and the mice were given aripiprazole (10 mg kg^−1^) or vehicle every 2 days, *n* = 6 mice/group. Bioluminescence and tumor images (F) of tumor growth in mice injected with indicated cells, including cells with LAMP2a‐deficiency, cells with re‐overexpression of LAMP2a‐wt or LAMP2a mut#1, and those treated with aripiprazole or vehicle, *n* = 6 mice/group. G) Hematoxylin and eosin staining of mouse intestines, *n* = 3 mice/group. H) Quantification of Ki‐67 proliferation index in tumors, *n* = 3 mice/group. Bars, SD; ^*^ *p* < 0.05; ^**^ *p* < 0.01; ^***^ *p* < 0.001; ns, no significant difference.

Molecular docking predicted that Leu394, Ile398, Lys401, and His402–404 (His402, His403, and His404) of LAMP2a are the most likely residues required for the binding of aripiprazole to it (**Figure** **4**A). We constructed three plasmids to express the different LAMP2a mutants: mut#1 (K401A, H402A, H403A, and H404A), mut#2 (L394A), and mut#3 (I398A). We overexpressed the mutants and wild‐type LAMP2a in LAMP2a‐deficient HT29 and DLD1 cells (Figure 4B). The sensitivity of LAMP2a‐deficient CRC cells to aripiprazole was markedly restored when wild‐type LAMP2a, mut#2, or mut#3 were re‐expressed at a level comparable to that of parental cells (Figure S6A, Supporting Information). Colony‐formation assay further confirmed that the antitumor effect of aripiprazole was restored in cells overexpressing wild‐type LAMP2a, but not in cells with mut#1 (Figure 4C). In addition, aripiprazole did not alter the levels of apoptotic or autophagic proteins in mut#1 cells (Figure S6B, Supporting Information). To confirm the direct binding of aripiprazole to the Lys401‐His404 of LAMP2a, the proteins of wild‐type LAMP2a and the mut#1 mutant were purified by means of binding titration with aripiprazole in vitro. Isothermal calorimetry showed that LAMP2a bound to aripiprazole with a binding constant of Kd = 1.3×10^−5^, while mutation of Lys401‐His404 on LAMP2a disrupted this binding effect (Figure 4D). It should be noted that such binding could increase the lysosomal content (Figure S6C, Supporting Information).

Furthermore, the role of LAMP2a in the anticancer properties of aripiprazole was evaluated using animal models. LAMP2a‐deficient DLD1‐Luc cells and cells overexpressing wild‐type LAMP2a and mut#1 were injected into the base of the cecum of nude mice in situ, to establish an orthotopic tumor model. Tumor growth was monitored using bioluminescence imaging. Each group was further divided into two subgroups for treatment with aripiprazole or the vehicle (Figure 4E). Consistent with the in vitro data, bioluminescence, and tumor images showed that aripiprazole did not inhibit the growth of tumors derived from LAMP2a‐knockout cells (Figure 4F). More importantly, the antitumor effect of aripiprazole was recovered when the cells were re‐overexpressed with wild‐type LAMP2a, but not with mut#1 (Figure 4F), which was further evidenced by histological analysis of xenografts (Figure 4G). In addition, aripiprazole significantly induced apoptosis in tumors overexpressing wild‐type LAMP2a, but not in those expressing mut#1 (Figure S6D, Supporting Information). We also observed that aripiprazole decreased the expression of the proliferation marker Ki‐67 and the autophagy marker p62 in the tumor, but increased the expression of LC3II (Figure 4H; Figure S6E, Supporting Information). Taken together, the Lys401‐His404 in LAMP2a are aripiprazole‐binding sites that are required for its anticancer bioactivity.

### 2.5 The RNH1/miR‐99a/mTOR Signaling Axis Mediates the Anticancer Effect of Aripiprazole

**Figure 5:**
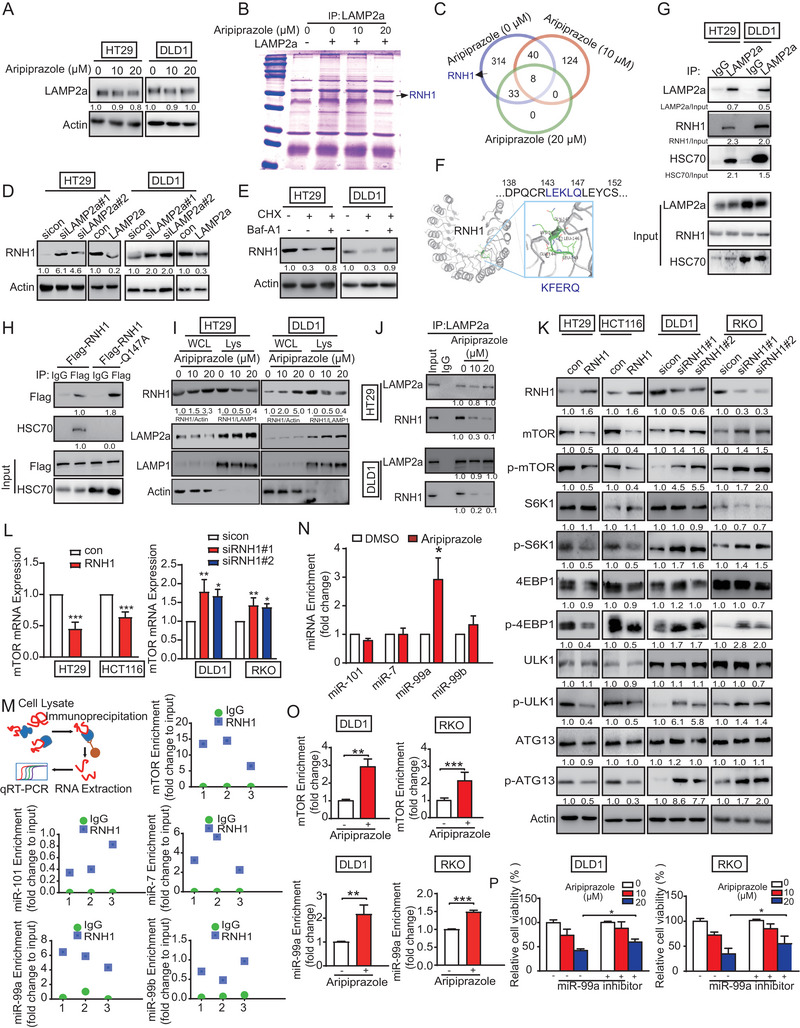
Aripiprazole suppresses mTOR through the RNH1/miR‐99a signaling axis. A) The expression of LAMP2a in HT29 and DLD1 cells treated with aripiprazole (10 and 20 µm) or dimethyl sulfoxide for 48 h was analyzed using western blot, *n* = 3/experiments. B) The binding partners of LAMP2a were identified using co‐immunoprecipitation coupled with mass spectrometry. Coomassie brilliant blue staining showing the specific bands with indicated treatment before mass spectrometry analysis, *n* = 3/experiments. C) Identification of overlapped proteins. D) The expression of RNH1 in CRC cells with LAMP2a knockdown and overexpression, *n* = 3/experiments. E) After pretreatment with cycloheximide (CHX; 50 µg mL^−1^) for 12 h, CRC cells were exposed to 10 nm Baf‐A1 and assessed for RNH1 expression using western blot, *n* = 3/experiments. F) A “KFERQ”‐like motif (LEKLQ) was found in the amino sequence of RNH1. G) The interactions of LAMP2a, HSC70, and RNH1 were determined using co‐immunoprecipitation, *n* = 3/experiments. H) The interaction between HSC70 and wild‐type or Q147A mutant RNH1 was determined using co‐immunoprecipitation, *n* = 3/experiments. I) RNH1 level in WCL (whole‐cell lysates) and Lys (purified lysosomes) of HT29 cells treated with various concentrations of aripiprazole (10 and 20 µm) or dimethyl sulfoxide for 48 h. LAMP2a (lysosome marker) and actin (cytosol marker) were examined to verify the enrichment of lysosomes, *n* = 3/experiments. J) Aripiprazole blocked the binding of LAMP2a and RNH1, *n* = 3/experiments. K,L) CRC cells were transfected with RNH1‐expressing plasmid or small interfering RNAs against RNH1, the protein expression levels of mTOR and its downstream phosphorylated proteins were determined using western blot (K), while the mRNA levels of mTOR were determined using RT‐qPCR (L), *n* = 3/experiments. M) The binding of RNH1 to mRNA of mTOR and miRNAs (miR‐101, miR‐7, miR‐99a, and miR‐99b) was detected by using RNA immunoprecipitation assay. Results have been shown as individual enrichment for each independent experiment, *n* = 3/experiments. N) Aripiprazole treatment (20 µM, 48 h) increased the binding of RNH1 to miR‐99a, *n* = 3/experiments. O) The enrichment of RNH1 with miR‐99a or mTOR mRNA was detected in CRC cells with aripiprazole treatment, *n* = 3/experiments. P) Silencing miR‐99a (50 nm) reversed the inhibitory effect of aripiprazole (48 h) on CRC cell proliferation, *n* = 3/experiments. Bars, SD; ^*^ *p* < 0.05; ^**^ *p* < 0.01; ^***^ *p* < 0.001; ns, no significant difference.

Next, we investigated the role of LAMP2a in aripiprazole‐induced mTOR inhibition. LAMP2a‐mediated CMA‐selected degradation of specific substrate is key for tumor progression,^[^ ^13^, ^15^, ^16^ ^]^ we therefore screened for the LAMP2a substrates that are responsible for aripiprazole‐induced mTOR and CRC inhibition. Initially, western blot confirmed that aripiprazole did not affect the protein level of LAMP2a (**Figure** **5**A). Then, we employed co‐immunoprecipitation (Co‐IP) coupled with mass spectroscopy (MS) to profile the substrates of LAMP2a (Figure 5B,C) in CRC cells treated with different concentrations of aripiprazole. In total, 351 (88.9%) proteins bearing KFERQ‐like motifs were identified. We found that 63.2% of these proteins accumulated in the aripiprazole‐treated group (Figure S7A, Supporting Information), suggesting that the binding of aripiprazole to specific amino acid residues in LAMP2a only partially inhibited CMA. Among the identified proteins, RNH1, which has been reported to regulate the expression of mTOR in breast cancer,^[^ ^18^ ^]^ was of interest. Next, we examined whether RNH1 was a substrate of LAMP2a. Western blot indicated that LAMP2a knockdown increased the expression of RNH1, while LAMP2a overexpression decreased it (Figure 5D). The effect of cycloheximide, which can inhibit the protein synthesis of RNH1 in CRC cells, was attenuated by treatment with the autophagy inhibitor Baf‐A1 (Figure 5E), suggesting that lysosomes may be involved in RNH1 degradation. HSC70 can recognize and interact with the substrates bearing a “KFERQ”‐like motif in their amino acid sequence, and LAMP2a serves as a receptor on the lysosome membrane for the HSC70‐substrate complex.^[^ ^12^ ^]^ Thus, we examined whether RNH1 contains a “KFERQ”‐like motif in its amino acid sequence. As displayed in Figure 5F, a pentapeptide “LEKLQ” (“KFERQ”‐like motif) was found at the positions 143–147 of RNH1, which is a putative selection signal for LAMP2a degradation. As expected, RNH1 and HSC70 co‐immunoprecipitated with LAMP2a (Figure 5G). To test whether the “LEKLQ” motif of RNH1 is essential for HSC70 binding, the amino acid “Q” was mutated to “A”. Co‐IP assays showed that the RNH1‐Q147A mutant, but not wild‐type RNH1, lost the ability to interact with HSC70 (Figure 5H).

In aripiprazole‐treated CRC cells, RNH1 accumulated in whole‐cell lysates, but decreased in purified lysosomes (Figure 5I). Aripiprazole treatment inhibited the binding of LAMP2a to RNH1 (Figure 5J). RNH1 is degraded via the ubiquitination‐proteasomal pathway.^[^ ^23^ ^]^ However, we found that the ubiquitination of RNH1 did not change in CRC cells after aripiprazole treatment (Figure S7B, Supporting Information). Moreover, western blot demonstrated that MG132, a proteasome inhibitor, did not dramatically promote the aripiprazole‐induced accumulation of RNH1 (Figure S7C, Supporting Information), suggesting that ubiquitination‐mediated RNH1 degradation was not involved in the anticancer function of aripiprazole. Furthermore, we established HSC70‐deficient CRC cells (Figure S7D, Supporting Information) and found reduced degradation of RNH1 in them (Figure S7E, Supporting Information), indicating that RNH1 is a substrate of LAMP2a upregulated by aripiprazole. We also analyzed the expression of known CMA substrates (PKM2, DJ‐1, and GAPDH) in aripiprazole‐treated CRC and LAMP2a‐knockdown cells (positive control). As shown in Figure S7F (Supporting Information), aripiprazole treatment induced the accumulation of RNH1, PKM2, DJ‐1, and GAPDH, similar to that induced by LAMP2a knockdown. Similar effects on the accumulation of RNH1 and the reduction of mTOR were observed in LAMP2a‐deficient and aripiprazole‐treated CRC cells (Figure S7G, Supporting Information). These results indicated that RNH1 is a key substrate of LAMP2a and is involved in the LAMP2a‐mediated mTOR signaling pathway.

It has been reported that RNH1 suppresses mTOR signaling by mediating mTOR mRNA decay.^[^ ^18^ ^]^ We found that the protein and mRNA expression levels of mTOR decreased upon RNH1 overexpression and increased upon RNH1 knockdown (Figure 5K,L). Thus, we speculated that RNH1 regulates miRNAs that target mTOR. A set of miRNAs, including miR‐101, miR‐7, miR‐99a, and miR‐99b, has been shown to target mTOR in various tumors. We performed RNA immunoprecipitation (RIP) assay and found that miR‐101, miR‐7, miR‐99a, and miR‐99b were specifically precipitated by RNH1 (Figure 5M; Figure S8A, Supporting Information). In particular, the binding between RNH1 and miR‐99a in CRC cells was enhanced following aripiprazole treatment (Figure 5N), suggesting that aripiprazole regulates mTOR levels by increasing the binding between miR‐99a and RNH1. We also found no significant changes in the expression levels of the other target genes (FGFR3, HOXA1, FAM64A, and IGF1R) of miR‐99a (Figure S8B,C, Supporting Information). These findings demonstrated that miR‐99a plays an important role in RNH1‐mediated mTOR mRNA decay.

In addition, western blot and RIP assay showed that aripiprazole increased the binding between RNH1, mTOR mRNA, and miR‐99a (Figure 5O). The WST‐1 assay indicated that treatment of miR‐99a with an inhibitor significantly abrogated the effect of aripiprazole on the proliferation of CRC cells (Figure 5P), suggesting that the RNH1/miR‐99a/mTOR regulatory axis is involved in the anticancer bioactivity of aripiprazole.

### 2.6 LAMP2a is Essential for the Bioactivity of Aripiprazole in the RNH1/miR‐99a/mTOR Regulatory Axis and Serves as a Favorable Prognostic Biomarker in CRC

**Figure 6:**
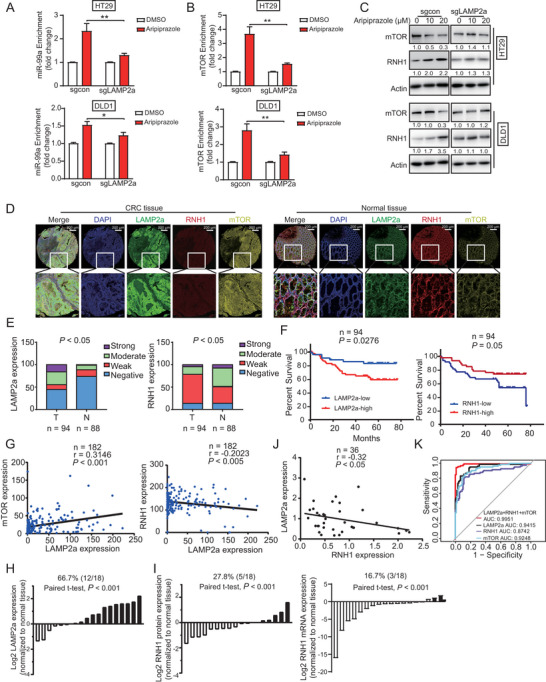
LAMP2a is essential for the bioactivity of aripiprazole in the RNH1/miR‐99a/mTOR regulatory axis and is a favorable prognostic biomarker for CRC. A,B) RIP assay was performed to determine the effect of aripiprazole (20 µm, 48 h) on the binding of RNH1 with miR‐99a (A) or mTOR mRNA (B) in HT29 and DLD1 cells, with or without LAMP2a‐deficency, *n* = 3/experiments. C) Western blot was performed to determine the effects of aripiprazole (0–20 µm, 48 h) on the expression of mTOR and RNH1 in LAMP2a‐deficent HT29 and DLD1 cells, *n* = 3/experiments. D) Representative immunohistochemistry images of LAMP2a, RNH1, and mTOR in CRC tumor (*n* = 94) and normal (*n* = 88) tissues. E) Comparison of LAMP2a and RNH1 expression in primary tumors and normal tissues. F) Kaplan–Meier analysis of CRC patients stratified according to tumor LAMP2a or RNH1 expression. G) The correlation between LAMP2a and RNH1 or mTOR expression in CRC tissues. H,I) The protein expressions of LAMP2a (H) and protein as well as mRNA expression of RNH1 (I) in 18 paired tissues (tumor tissues and corresponding adjacent normal tissues) were determined using western blot, and are shown in the form of a graph. J) The correlation between LAMP2a and RNH1 expression in CRC tissues. K) Receiver operating characteristic curve analysis of CRC (GSE37182). Bars, SD; ^*^ *p* < 0.05; and ^**^ *p* < 0.01.

As mentioned above, LAMP2a knockout markedly weakened the inhibitory effects of aripiprazole on CRC (Figure 3). Next, the effect of aripiprazole on RNH1/miR‐99a/mTOR signaling was determined in LAMP2a‐deficient CRC cells. As shown in **Figure** **6**A,B, unlike the significant effects on HT29 and DLD1 parental cells, aripiprazole treatment did not alter the binding between mTOR mRNA, miR‐99a, and RNH1 in LAMP2a‐deficient CRC cells. Aripiprazole also did not change the expression of RNH1 and mTOR when LAMP2a was silenced (Figure 6C). These observations indicated that LAMP2a is required for aripiprazole‐induced RNH1/miR‐99a/mTOR regulatory axis in CRC.

To study the clinical relevance of LAMP2a and RNH1, we detected the expression of LAMP2a, RNH1, and mTOR in a tissue microarray containing 94 CRC tumor and 88 normal tissue samples, by means of multiplex immunohistochemistry staining. As shown in Figure 6D,E, most tumor tissues had higher LAMP2a expression than adjacent normal tissues, and 80% of tumor tissues displayed low RNH1 expression (negative and weak), whereas only 50% of normal tissues displayed low RNH1 expression. Kaplan–Meier survival analysis revealed shorter survival in patients with CRC who displayed high LAMP2a expression than in those with low LAMP2a expression, and patients with CRC who displayed lower RNH1 expression had significantly shorter survival than those with higher RNH1 expression (Figure 6F). Notably, LAMP2a expression correlated negatively with RNH1 expression and positively with mTOR expression (Figure 6G).

In addition, we analyzed 18 pairs of CRC tumor and adjacent normal tissues to determine the clinical significance of LAMP2a and RNH1 expression using western blot. The majority of tumor cases had a stronger expression of LAMP2a (66.7%) in tumors than in adjacent normal tissues (Figure 6H; Figure S8D, Supporting Information), and only 27.5% and 16.7% of tumors had stronger protein and mRNA expression of RNH1 than adjacent normal tissues (Figure 6I), respectively. In addition, LAMP2a expression correlated positively with RNH1 expression (Figure 6J). Moreover, the receiver operating characteristic curve illustrated that LAMP2a, RNH1, and mTOR could be used as diagnostic markers for CRC, with area under the curve values of 0.9415, 0.8742, and 0.9248, respectively (Figure 6K). Importantly, the cooperative units of the three genes may serve as a novel cluster of prognostic biomarkers (area under the curve = 0.9951). This result was further confirmed by receiver operating characteristic curve analysis of CRC, breast adenocarcinoma, and hepatocellular carcinoma cohorts (GSE198692, GSE70947, GSE84005; Figure S8E, Supporting Information). Collectively, these results suggested that the cooperative units of LAMP2a, RNH1, and mTOR may provide a technically simple approach for prognostic diagnosis of CRC.

### 2.7 Aripiprazole Enhances the Sensitivity of CRC Cells to 5‐FU

**Figure 7:**
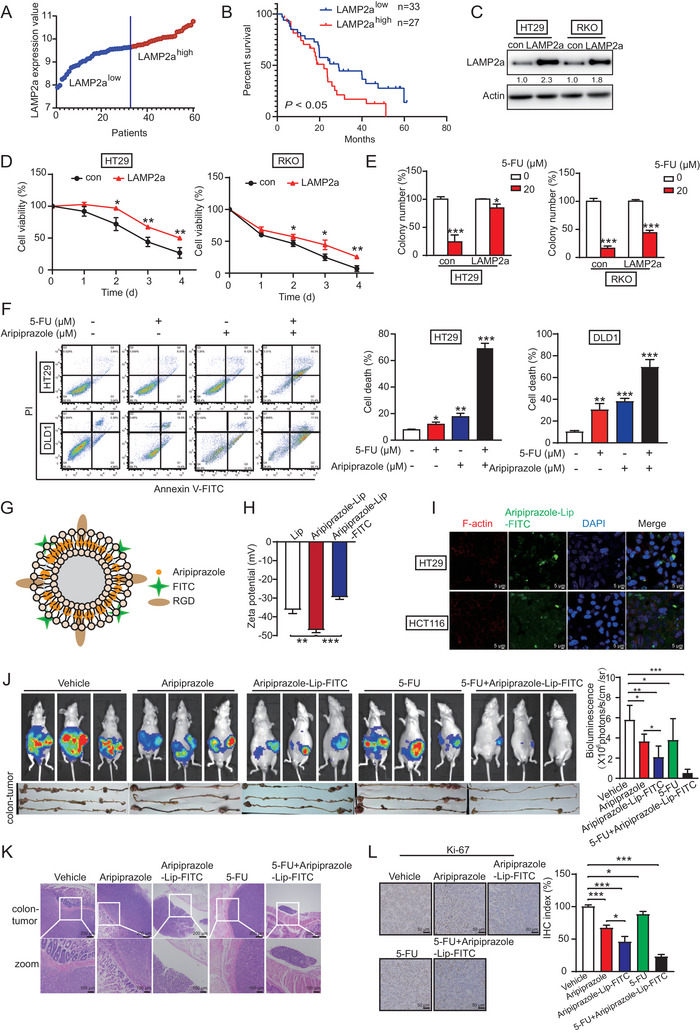
Aripiprazole enhances the sensitivity of CRC cells to 5‐FU. A,B) The StepMiner algorithm was used to stratify the 60 CRC patients received 5‐FU‐based adjuvant chemotherapy (FOLFIRI, GSE72970) into LAMP2a‐high (*n* = 27) and –low (*n* = 33) subgroups (A), and the association of LAMP2a with prognosis and benefit from 5‐FU‐based adjuvant chemotherapy was evaluated (B). C–E) Both CRC cells transfected with LAMP2a‐expressing plasmid or vector control (C) were treated with 5‐FU (20 µM) for 48 h, and the cell viability and proliferation were compared by means of WST‐1 (D) and colony‐formation (E) assays, respectively, *n* = 3/experiments. F) Cell apoptosis in CRC cells treated with 5‐FU (10 µm, 48 h), aripiprazole (10 µm, 48 h) alone, or the combination of 5‐FU and aripiprazole were determined using the Annexin V/propidium iodide assay, *n* = 3/experiments. G) Structure of aripiprazole packaged by the FITC‐labeled liposome. H) Zeta potential values, *n* = 3/experiments. I) The uptake of aripiprazole‐liposome‐FITC by CRC cells was determined using confocal assay, *n* = 3/experiments. J) Bioluminescence (upper panel) and tumor (lower panel) images of tumor growth in mice injected with DLD1‐Luc cells and treated with vehicle, aripiprazole (5 mg kg^−1^), aripiprazole‐liposome‐FITC (5 mg kg^−1^), 5‐FU (10 mg kg^−1^), or the combination of aripiprazole‐liposome‐FITC and 5‐FU, *n* = 6 mice/group. K) Hematoxylin and eosin staining of mouse intestines, *n* = 3 mice/group. L) Quantification of Ki‐67 proliferation index in tumors, *n* = 3 mice/group. Bars, SD; ^*^ *p* < 0.05; ^**^ *p* < 0.01; ^***^ *p* < 0.001; ns, no significant difference.

Chemotherapy resistance frequently leads to poor prognosis of patients with CRC. To explore whether the sensitivity of CRC to chemotherapy can be regulated by LAMP2a, we divided the 60 CRC patients received 5‐FU‐based adjuvant chemotherapy (GSE72970) into LAMP2a‐high and ‐low groups, according to LAMP2a expression (**Figure** **7**A). Patients with LAMP2a‐high had shorter survival than those with LAMP2a‐low, suggesting that patients with LAMP2a‐low benefited significantly from 5‐FU‐based adjuvant chemotherapy (*P* < 0.05, Figure 7B). Therefore, we investigated the effect of LAMP2a on the chemotherapeutic response of CRC by establishing LAMP2a‐overexpressing CRC cells (Figure 7C). WST‐1 and colony‐formation assays showed that LAMP2a overexpression significantly reversed the anticancer effects of 5‐FU (Figure 7D,E). Moreover, a combination of low‐dose aripiprazole (10 µM) with low‐dose 5‐FU (10 µM) significantly suppressed cell proliferation and promoted apoptosis, as compared to aripiprazole or 5‐FU alone (Figure 7F; Figure S9A, Supporting Information).

To enhance the treatment effect of aripiprazole on CRC, we carried aripiprazole on a suitable vehicle, liposomes labeled with FITC and arginine‐glycine‐aspartic acid peptides, to achieve tumor specificity (Figure 7G). The zeta potential of liposome, aripiprazole‐liposome, and aripiprazole‐liposome‐FITC were −35 ± 2, −48 ± 1, and −28 ± 1.5 mV, respectively (Figure 7H). The ultraviolet‐visible spectrum showed that the absorption peak of aripiprazole‐liposome and aripiprazole‐liposome‐FITC was at 225 nm, and the encapsulation efficiency was 79.5% (Figure S9B, Supporting Information), suggesting successful preparation of aripiprazole‐liposome‐FITC. Confocal microscopy showed that aripiprazole‐carrying liposomes could be absorbed by CRC cells (Figure 7I). As above, CRC cells DLD1‐Luc were injected into the base of the cecum of nude mice in situ, to establish an orthotopic tumor model. Nude mice were divided into five subgroups and intraperitoneally injected with vehicle, aripiprazole, aripiprazole‐liposome‐FITC, 5‐FU, or a combination of aripiprazole‐liposome‐FITC and 5‐FU. Bioluminescence and tumor images showed that aripiprazole‐liposome inhibited tumor growth more significantly than aripiprazole alone, and the combination of aripiprazole‐liposome and 5‐FU exerted even more suppressive effect on tumor growth than the control and single treatment groups (Figure 7J). This effect was further evidenced by hematoxylin and eosin staining (Figure 7K) and quantified in terms of the Ki‐67 proliferation index (Figure 7L) of the tumors. We also noted no significant differences in body weight (Figure S9C, Supporting Information), critical organ histological features (Figure S9D, Supporting Information), or serum levels of aspartate and alanine aminotransferases (Figure S9E, Supporting Information). Collectively, these data demonstrated that aripiprazole can increase the sensitivity of CRC cells to 5‐FU, and may thus, serve as a 5‐FU sensitizer in CRC with low toxicity.

## 3 Discussion

CRC remains one of the most malignant tumors with poor prognostic outcomes that urgently needs efficient therapeutic agents that can improve treatment. In this regard, drug repurposing provides a quick and economical strategy to search for new anticancer drugs. Because mTOR signaling plays a crucial role in sustaining cancer survival,^[^ ^19^, ^20^, ^21^ ^]^ drugs that target this signaling pathway have achieved significant anticancer effects in the clinic. For example, everolimus, a rapamycin analog, has been used to treat cancer by suppressing mTOR signaling.^[^ ^22^, ^23^ ^]^ In this study, we repurposed aripiprazole, an atypical antipsychotic drug, as an effective anticancer agent for CRC, which targets LAMP2a to induce RNH1/miR‐99a/mTOR‐mediated autophagy and subsequent apoptosis.

**Figure 8:**
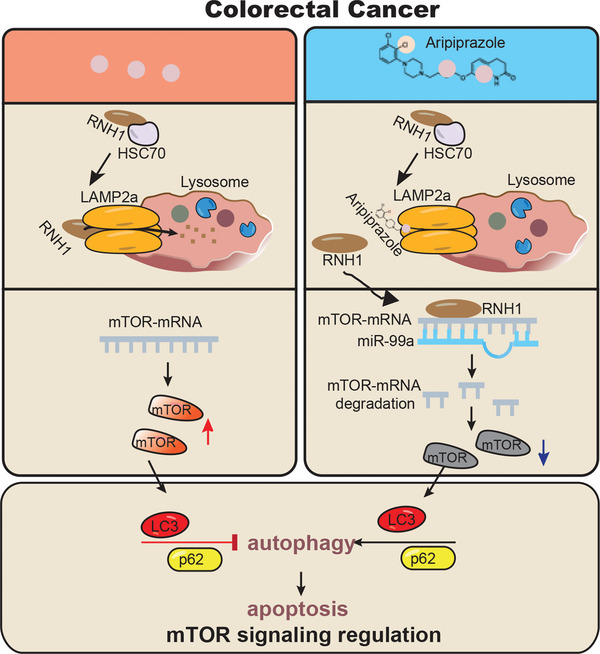
Schematic diagram illustrating how aripiprazole suppresses colorectal tumorigenesis. Aripiprazole directly binds LAMP2a to prevent the HSC70‐mediated lysosomal degradation of RNH1, inactivating RNH1/miR‐99a/mTOR signaling, thereby suppressing CRC tumorigenesis via autophagy‐mediated apoptosis.

Aripiprazole has been reported to suppress tumor growth by targeting Gαq and HTR1A, and therefore inhibiting PI3K/AKT/mTOR signaling.^[^ ^24^, ^25^ ^]^ Indeed, mTOR inhibition by aripiprazole was also observed in this study, in which we identified LAMP2a as a novel target. Direct binding of aripiprazole‐LAMP2a prevented the HSC70‐mediated lysosomal degradation of RNH1 and inactivated RNH1/miR‐99a/mTOR signaling, thereby suppressing CRC tumorigenesis via autophagy‐mediated apoptosis (**Figure** **8**). The anticancer effect of aripiprazole on CRC was achieved when HTR1A, the original target of aripiprazole, was deleted, whereas this effect was restored when LAMP2a was deficient, suggesting that LAMP2a is the key molecule for the anticancer effect of aripiprazole.

LAMP2a, a highly glycosylated transmembrane protein mainly located on the surface of lysosomes, regulates the rate‐limiting step of CMA.^[^ ^26^, ^27^ ^]^ Recent studies have demonstrated that the levels of LAMP2a and related CMA activities are significantly increased in various cancer cells.^[^ ^13^, ^15^, ^16^ ^]^ Blocking CMA activity or LAMP2a expression effectively reduced the proliferation and metastasis of malignant cells, suggesting the potential of CMA or LAMP2a inhibitors in anticancer therapy.^[^ ^28^, ^29^ ^]^ To date, silencing LAMP2a remains the major strategy to inhibit CMA, and there is not much clarity on the chemical inhibitors of CMA.^[^ ^30^ ^]^ In this study, we identified LAMP2 as a direct target of aripiprazole using a DARTS assay. We further determined that LAMP2a, but not the other variants (LAMP2b and LAMP2c), mediated the anticancer effect of aripiprazole (Figure 3), as evidenced by the fact that LAMP2a knockout‐mediated CMA deficiency significantly weakened the bioactivity of aripiprazole (Figure 6). We further characterized that aripiprazole binds to Lys401‐His404 of LAMP2a, a unique region in LAMP2a that is distinct from LAMP2b and LAMP2c. Therefore, only LAMP2a re‐expression mediated the anticancer effects of aripiprazole in vivo and in vitro (Figure 4). Moreover, the binding of aripiprazole to the cytoplasmic tail of LAMP2a did not influence the lysosomal content. This is the first study to report aripiprazole as a novel CMA inhibitor that suppresses CRC.

Many studies have documented that there is a crosstalk of LAMP2a‐mediated CMA with macroautophagy,^[^ ^31^, ^32^ ^]^ however, the underlying mechanism remains to be elucidated. It is estimated that many soluble cytosolic proteins containing the KFERQ motif are involved in LAMP2a‐selective autophagy.^[^ ^33^ ^]^ Canonical KFERQ‐like motifs are always flanked by a glutamine on one of the sides (Q) and contain one or two of the positive residues K and R, one or two of the hydrophobic residues F, L, I, or V, and one of the negatively charged E or D residues.^[^ ^33^ ^]^ Consistently, our Co‐IP‐MS profiling of LAMP2a substrates on CRC cells identified 278 (89%) proteins bearing a KFERQ‐like motif, 63.2% of which were enriched in the aripiprazole‐treated group (Figure S7A, Supporting Information), indicating that the binding of aripiprazole to specific amino acid residues in LAMP2a partially inhibited CMA. Among these predicted CMA substrates, there was significant accumulation of RNH1, a protein functionally relevant to mTOR and bearing the KFERQ‐like motif (LEKLQ), in the aripiprazole‐treated group (Figure 5B,H), which was therefore, selected for subsequent investigation. Meanwhile, RNH1 expression (both mRNA and protein levels) is frequently downregulated in CRC and correlates with poor prognosis in patients with CRC, suggesting a tumor‐suppressive role of RNH1 in CRC. We also found that RNH1 formed a complex with miR‐99a to induce mTOR mRNA degradation, thereby promoting autophagy. This finding provides a rational link between LAMP2a‐mediated CMA and macroautophagy, in which high CMA activity drives the lysosomal degradation of RNH1, leading to mTOR activation and macroautophagy inhibition. Many apoptotic stimulators can trigger macroautophagy, a process that usually appears before cell apoptosis.^[^ ^34^ ^]^ We verified that, aripiprazole‐triggered apoptosis could be attenuated by pretreatment with Baf‐A1, while aripiprazole‐induced autophagy could not be restored by Z‐VAD‐FAM treatment (Figure S4A–E, Supporting Information), suggesting that autophagy is required for aripiprazole‐induced apoptosis.

5‐FU‐based chemotherapy is widely used for cancer treatment in the clinic; however, the acquisition of resistance to 5‐FU remains a major problem.^[^ ^35^, ^36^ ^]^ Inhibition of CMA or LAMP2a has recently been reported to sensitize tumor cells to a variety of anticancer agents.^[^ ^33^ ^]^ In this study, we found that high expression of LAMP2a is the cause of resistance of CRC cells to 5‐FU and that aripiprazole can bind LAMP2a to block its activity, thus increasing the sensitivity to 5‐FU. This significant synergistic effect inspired us to employ liposomes labeled with FITC and arginine‐glycine‐aspartic acid as delivery vehicles for efficient localization and selective release of aripiprazole. Our in vivo experimental results validated that aripiprazole packaged in liposomes displayed a strong anticancer effect, which was amplified upon combined treatment with 5‐FU. This is the first study to prove that antipsychotic drug aripiprazole, as a single agent or in combination with 5‐FU, can significantly induce autophagy‐mediated apoptosis to inhibit tumorigenesis of CRC. By regulating the LAMP2a/RNH1/miR‐99a/mTOR signaling axis to make anti‐cancer effects, aripiprazole administration may be an efficient strategy for CRC therapy.

## 4 Experimental Section

### Cell Culture and Drugs

Human CRC cell lines HT29, DLD1, HCT116, and RKO were purchased from the American Type Culture Collection (Rockville, MD, USA). NCM460 cells were obtained from INCELL (San Antonio, TX, USA). Roswell Park Memorial Institute‐1640 (Thermo Fisher Scientific, Waltham, MA, USA) supplemented with 10% fetal bovine serum (ExCell Bio, Shanghai, China) was used to culture the CRC cell lines in an incubator containing 5% CO_2_, at 37 °C. Aripiprazole and Baf‐A1 were purchased from Selleck (Houston, TX, USA) and dissolved in dimethyl sulfoxide. 5‐FU was obtained from Topscience (Shanghai, China) and dissolved in water.

### Cell Viability Assay

The WST‐1 Cell Proliferation and Cytotoxicity Assay Kit (Beyotime Biotechnology, Shanghai, China) was used to measure cell viability, as described previously.^[^ ^10^ ^]^ CRC cells were seeded into 96‐well plates and treated with different drug concentrations. Following that, WST‐1 was added to the plate and incubated with the cells for 2 h, at 37 °C. Absorbance at the wavelength of 450 nm was measured using an automated microplate spectrophotometer (BioTek Instruments, Winooski, VT, USA).

### Colony‐Formation Assay

CRC cells subjected to different treatments were seeded into 6‐ or 12‐well plates and cultured for two weeks. The cells were then washed three times with phosphate‐buffered saline (PBS), fixed with 75% ethanol for 10 min, and stained with 1% crystal violet for 10 min. Finally, the number of colonies formed was counted.

### Annexin V‐FITC/PI Staining Assay

Cell apoptosis was measured using an Annexin V‐FITC/PI Apoptosis Detection Kit (KeyGen, Nanjing, Jiangsu, China), according to the manufacturer's instructions. The collected cells were washed twice with PBS, suspended in binding buffer, and stained with Annexin V‐FITC and PI for 10 min, at room temperature, in the dark. A BD FACSCelesta™ flow cytometer (BD Biosciences, San Diego, CA, USA) was used to analyze the apoptotic cells.

### Western Blot

Clinical specimens were obtained from the First Affiliated Hospital of Jinan University, and the experiments were approved by the Ethics Committee of Jinan University (approval no. KY‐2022‐197). The preparation of cell lysates and the protocol for western blot have been described previously.^[^ ^37^, ^38^ ^]^ The antibodies used included those against LC3, p62, RNH1, GAPDH, PGK, LAMP2, LAMP1, and HSC70 from ProteinTech (Chicago, IL, USA), Caspase‐3, cleaved Caspase‐3, p‐mTOR, mTOR, p‐ATG13, p‐ULK1, p‐4EBP1, and p‐S6K1 from Cell Signaling Technology (Beverly, MA, USA); LAMP2a from Abcam (Cambridge, MA, USA); and actin from Santa Cruz Biotechnology (Santa Cruz, CA, USA). Signals were detected using the Clarity™ Western ECL Substrate Kit (Bio‐Rad, Hercules, CA, USA).

### Co‐IP

Cells were collected in IP lysis buffer (Cell Signaling Technology), following which the lysates were incubated with IgG (Santa Cruz Biotechnology) and protein A/G Sepharose beads (Invitrogen, Gaithersburg, MD, USA), at 4 °C, for 1 h. The supernatant obtained was collected and incubated with primary antibodies at 4 °C overnight, following which A/G Sepharose beads were added and incubated for 4 h. The beads were washed three times with PBS and IP lysis buffer and then mixed with loading buffer for western blot analysis.

### MS and Bioinformatics Analyses

Protein digestion and MS were performed as previously described.^[^ ^10^ ^]^ Cells were lysed with sodium dodecyl sulfate lysis buffer (Beyotime Biotechnology). Proteins were digested with trypsin (Promega, Fitchburg, WI, USA), vacuum freeze‐dried, resuspended in anhydrous acetonitrile, and desalted using a MonoTIP™ C18 pipette tip (GL Sciences, Tokyo, Japan). The peptide samples were analyzed using an Orbitrap Fusion™ Lumos™ mass spectrometer (Thermo Fisher Scientific). The generated raw data were subjected to Proteome Discoverer 2.4 software (Thermo Fisher Scientific) for database searching. Protein and peptide false discovery rates were set at 1%. Ingenuity Pathway Analysis (Ingenuity Systems, Redwood City, CA, USA) was used to analyze the differentially expressed proteins (DEPs).

### TUNEL

Apoptosis was detected using the TUNEL in situ Cell Death Detection Kit, fluorescein (Roche Diagnostics, Mannheim, Germany). Slides were deparaffinized, rehydrated, and incubated with the TUNEL reaction mixture for 1 h, followed by 4′,6‐diamidino‐2‐phenylindole (DAPI) staining. The percentage of apoptotic cells was determined.

### Plasmids, Transfection, Infection, and CRISPR/Cas9‐Mediated Gene Knockout

Full‐length LAMP2a was amplified and cloned into the prokaryotic expression plasmid, pET‐28b (Novagen, Madison, WI, USA). Small interfering RNAs against RNH1, stable RNH1‐overexpressing plasmids, and plasmids expressing short hairpin RNA against RNH1 were purchased from TransheepBio (Shanghai, China). mTOR‐flag‐overexpressing plasmids and plasmids expressing single guide RNAs against LAMP2, LAMP2a, LAMP2b, and LAMP2c were obtained from IGEbio (Guangzhou, China). Using ClonExpress II One‐Step Cloning Kit (Vazyme, Nanjing, China), the plasmid pLenti CMV GFP Hygro‐LAMP2a‐wt was generated. The mutant constructs for stable LAMP2a‐mut1‐ (K401A, H402A, H403A, and H404A), LAMP2a‐mut2‐ (L394A), LAMP2a‐mut3‐ (I398A), and pET28b‐LAMP2a‐mut1‐ (K401A, H402A, H403A, and H404A) overexpressing plasmids were created using the Fast MultiSite Mutagenesis System (TransGen Biotech, Beijing, China). miR‐99a inhibitor (5′‐ CACAAGAUCGGAUCUACGGGUU‐3′) was purchased from RiboBio (Guangzhou, China). Transfection and establishment of stable cell lines were performed as described previously.^[^ ^39^ ^]^ The primer sequences used for cloning and generating mutations, as well as single guide RNA and small interfering RNA sequences used in this study are listed in Table S1 (Supporting Information).

### TEM

The cells were fixed with a TEM fixative (Wuhan Servicebio Technology, Wuhan, China), at 4 °C for 4 h, pre‐embedded in 1% agarose, and fixed with 1% osmium tetroxide. After dehydration at room temperature using ethanol, the cells were embedded in Poly/Bed 812 resin, followed by polymerization at 65 °C. Ultrathin sections were stained with 2% uranium acetate‐saturated alcohol solution and 2.6% lead citrate. A transmission electron microscope (HT7700, Hitachi, Fukuoka, Japan) was used to obtain images for morphological analyses.

### Immunofluorescence

The cells were washed thrice with PBS, fixed with 4% paraformaldehyde at room temperature for 15 min, and permeabilized with 0.1% Trito X‐100 at room temperature for 15 min. Next, the cells were incubated with primary antibodies at 4 °C overnight, after blocking with 5% bovine serum albumin at room temperature, for 1 h. The samples were then treated with the corresponding fluorescent secondary antibodies at room temperature for 2 h, in the dark, followed by staining with F‐actin or DAPI (Thermo Fisher Scientific). Images were obtained using a laser scanning confocal microscope (Carl Zeiss AG, Jena, Thuringia, Germany) and analyzed.

### Measurement of Lysosome Content

Cells were plated in 6‐well plates (4 × 10^5^ cells well^−1^) and treated with aripiprazole (0, 10, or 20 µM), then digested with trypsin and rinsed using PBS buffer. The cells were suspended in fetal bovine serum‐free Roswell Park Memorial Institute‐1640 medium and stained with LysoTracker™ Red (Beyotime Biotechnology) for 30 min, at 37 °C, away from light. The cells were then rinsed twice with PBS and analyzed using a BD FACSCelesta™ flow cytometer.

### Multiplex Immunohistochemical Staining

The PANO 6‐plex Immunohistochemistry Kit (Panovue, Beijing, China) was used to analyze LAMP2a, RNH1, and mTOR expression and their correlation with clinicopathological parameters in a tissue microarray containing 94 CRC tissue samples and 88 matched adjacent normal tissues (Shanghai Outdo Biotech, Shanghai, China). Briefly, the slides were deparaffinized in xylene, rehydrated in ethanol, blocked with blocking buffer (Panovue), incubated with primary antibodies for 60 min, washed with TBST buffer, and incubated with horseradish peroxidase‐conjugated secondary antibodies for 10 min. The slides were incubated with primary antibodies and visualized using Opal 520 TSA (1:200 dilution), followed by DAPI staining for 10 min. A Vectra Polaris automated pathology imaging platform (PerkinElmer, Waltham, MA, USA) was used to acquire images, which were analyzed using the HALO digital pathology analysis platform (Indica Labs, Corrales, NM, USA). A scale of negative to weak, representing low expression, and a scale of moderate to strong, representing high expression, was used to grade the intensity of LAMP2a, RNH1, and mTOR staining.

### Immunohistochemistry

In brief, sample slides were blocked with normal serum and then incubated with anti‐Ki‐67 (Dako, Mississauga, ON, Canada), ‐p62, or ‐LC3II antibodies overnight, at 4 °C, followed by incubation with corresponding biotinylated secondary antibodies and peroxidase‐conjugated avidin‐biotin complex (Dako). 3,3′‐diaminobenzidine (Dako) was used as the chromogen for immunostaining, and the sections were counterstained with hematoxylin. A Vectra^®^ Polaris automated pathology imaging platform (PerkinElmer) was used to acquire the images, which were analyzed using the HALO digital pathology analysis platform.

### Lysosome Isolation

Cells were washed with PBS, suspended in homogenization buffer (0.25 M sucrose, 2 mM EDTA, 10 mM HEPES, pH 7.4), and then centrifuged at 800 × *g* for 15 min. The supernatant was centrifuged at 6800 × *g* for 15 min and then centrifuged at 25000 × *g* for 10 min. The precipitate was layered in Percoll solution (Sigma‐Aldrich, St. Louis, MO, USA) and centrifuged at 35000 × *g* for 2 h. Lysosomes were collected, centrifuged at 100000 × *g* for 1 h, and then washed with PBS at 18000 × *g* for 30 min. LAMP2a, LAMP1, and actin were used to analyze the purity of lysosomes, and all steps were performed at 4 °C.

### RIP Assay

The interactions between RNH1, mTOR mRNA, and miR‐99a were analyzed using an RIP Kit (Suyan Technology, Guangzhou, China), according to the manufacturer's protocols. Briefly, cells were collected, lysed, and incubated with RIP buffer containing magnetic beads conjugated to anti‐RNH1, with IgG as a negative control. Following that, RNA was extracted from the samples and analyzed using quantitative real‐time PCR (RT‐qPCR).

### RT‐qPCR Analysis

Total RNA was extracted using TRIzol and reverse transcription was performed using the PrimeScript II First Strand cDNA Synthesis Kit (Takara, Dalian, China). SYBR Premix Ex Taq II (Takara) was used to analyze the mRNA expression of genes and the internal control, GAPDH, using a MiniOpticon real‐time PCR system (Bio‐Rad). The expression levels of miRNAs and U6 (as an internal control) were detected with the TaqMan microRNA assay (Thermo Fisher Scientific). The primer sequences used for RT‐qPCR are listed in Table S1 (Supporting Information).

### DARTS

DARTS was performed as described previously.^[^ ^10^ ^]^ Briefly, aripiprazole (20 µM) or dimethyl sulfoxide was added to the cell lysates, and incubated for 3 h at room temperature. Protease K (1 µL, 20 mg mL^−1^) was added to the cell lysates for 30 min and the samples were analyzed by means of western blot and stained with Coomassie Blue. Finally, the bands were analyzed using MS.

### Purification of LAMP2a Protein

Plasmids pET‐28b‐LAMP2a wt and pET‐28b‐LAMP2a mut#1, expressing wild‐type and mutant histidine (His)‐tagged LAMP2a fusion proteins, respectively, were constructed and transformed into *E. coli* BL21 (DE3) star cells. After the optical density at the wavelength of 600 nm of the bacterial culture reached ≈ 0.6, 0.5 mM isopropyl β‐D‐thiogalactopyranoside was added to the culture and incubated for 12 h, at 37 °C. A His‐tag protein purification kit (Beyotime Biotechnology) was used to isolate the His‐tagged fusion protein, which was analyzed using a western blot.

### Isothermal Titration Calorimetry

The binding of aripiprazole to LAMP2a was analyzed using a MicroCalorimeter Auto‐ITC 200 (Malvern, UK). Aripiprazole (200 µM in PBS) was titrated into the recombinant LAMP2a (20 µM in PBS) solution. Aripiprazole alone was used as a reference. Titration parameters: 2 µL of aripiprazole solution was injected into the 200‐µL protein sample cell. The delay between injections was 3 min and the stirring speed was 750 rpm.

### Molecular Docking

Protein databank (PDB; http://www.rcsb.org) was used to construct the LAMP2a protein model. The pose with the best score for aripiprazole and LAMP2a binding was selected for interaction analysis using Dock version 6.9 (https://dock.compbio.ucsf.edu/). Finally, PyMol (https://pymol.org/edu/) was used to perform aripiprazole‐LAMP2a interaction analysis and draw the image of the docked pose.

### Synthesis and Characterization of Aripiprazole‐Liposome‐FITC

PC (15 mg), cholesterol (2.5 mg), and aripiprazole were dissolved in 100 mL anhydrous ethanol and stirred for 20 min to obtain a colloid solution. Next, 1 mL of the aripiprazole‐liposome nanosystem was obtained by resuspending the concentrated solution in PBS (pH 7.4). Subsequently, 1 mg mL^−1^ FITC and aripiprazole‐liposome were mixed (volume ratio, 1:1) and stirred overnight at room temperature, in the dark. Finally, the resulting aripiprazole‐liposome‐FITC nanoparticles were resuspended in PBS (pH 7.4) and centrifuged. The size distribution and morphology of the samples were measured using dynamic light scattering (Malvern; Zetasizer Nano ZS). The ultraviolet‐visible absorbance was determined using a spectroscope.

### Tumor Xenograft Experiments

Female BALB/c nude mice aged 6–8 weeks were maintained under standard conditions and cared for according to the institutional guidelines for animal care. All animal experiments were approved by the Ethics Committee for Animal Experiments at the Jinan University (20210630‐03). All animal work was performed in strict accordance with the approved protocol. CRC cells were digested and resuspended in PBS, mixed with an equal volume of Matrigel (BD Biosciences), and subcutaneously injected into the flanks of nude mice. When the tumor size reached 5 mm, the mice were randomly divided into treatment and control groups, which received oral gavage of aripiprazole and vehicle (0.5% sodium carboxymethyl cellulose, CMC‐Na), respectively, every 2 d. Body weight and tumor size were measured [V = (length×width^2^) / 2) every 2 d. Finally, the tumors and major organs were collected for histological, immunohistochemical, and western blot analyses. A commercial kit (HuiLi Biotech, Changchun, China) was used to analyze alanine and aspartate aminotransferase levels in the mouse serum.

### Orthotopic Tumor Model

CRC cells resuspended in PBS were injected into the base of the cecum of nude mice. After three weeks, the mice were randomly divided into treatment and control groups, which received oral gavage of aripiprazole and vehicle, respectively, every 2 d. Bioluminescence imaging was performed to observe the tumors in the mice (Xenogen IVIS Lumima II, PerkinElmer), and the signal was analyzed using Living Image R Software version 3.1. Tumors, major organs, and serum samples were collected for further analyses.

### Statistical Analysis

All in vitro experiments were performed in triplicate. Prism software (GraphPad) was used to perform two‐tailed Student's *t*‐test, one‐ and two‐way analysis of variance, and log‐rank test analyses. The values are presented as mean ± SD. The datasets used to analyze the receiver operating characteristic curve were downloaded from GEO (GSE37182, GSE198692, GSE70947, and GSE84005). The GEO database was also used to analyze the pathways involved in CRC (GSE115313) and the association between LAMP2a expression and patient survival (GSE72970). Survival was analyzed using the Kaplan–Meier method and log‐rank test. Bars, SD; ^*^ *p* < 0.05; ^**^ *p* < 0.01; ^***^ *p* < 0.001; ns, no significant difference.

### Ethics Approval and Consent to Participate

This work was approved by the Ethics Committee of Jinan University. All animal experiments were approved by the Ethics Committee for Animal Experiments at the Jinan University (20210630‐03).

## Conflict of Interest

The authors declare no conflict of interest.

## Author Contributions

H.‐F.H., J.‐Y.F., and L.H. contributed equally to this work. H.F.H. performed the in vitro experiments, data analysis, and drafted the manuscript; H.F.H., J.Y.F., L.H., and G.B.G. performed the in vivo experiments; J.Y.F., L.H., W.X.Z., N.L., Y.J.L., Y.F.L., X.F.D. were involved in the experiments and statistical analysis; S.M.Y. assisted in the synthesis and characterization of aripiprazole‐liposome‐FITC; Y.L.P. and Y.W. provided the technical support and advice; H.F.H. and Q.Y.H. were involved in the conception and design of the study, as well as manuscript revisions. All the authors have read and approved the final version of the manuscript.

## Supporting information



Supporting Information

Supplemental Table 1

Supplemental Table 2

Supplemental Table 3

## Data Availability

The data that support the findings of this study are available in the supplementary material of this article.
