# Supplementary material for: The Antipsychotic Drug Aripiprazole Suppresses Colorectal Cancer by Targeting LAMP2a to Induce RNH1/miR‐99a/mTOR‐Mediated Autophagy and Apoptosis
Source: Adv Sci (Weinh). 2024 Nov 8;11(48):2409498. doi: 10.1002/advs.202409498 (PMC11672294; doi:10.1002/advs.202409498)
Supplement: Supplementary file 1 — Supporting Information [file ADVS-11-2409498-s003.pdf]

ADVANCED SCIENCE
Open Access

Supporting Information

for *Adv. Sci.*, DOI 10.1002/advs.202409498

The Antipsychotic Drug Aripiprazole Suppresses Colorectal Cancer by Targeting LAMP2a to Induce RNH1/miR-99a/mTOR-Mediated Autophagy and Apoptosis

Hui-Fang Hu, Jia-Ying Fu, Lei Han, Gui-Bin Gao, Wei-Xia Zhang, Si-Ming Yu, Nan Li, Yang-Jia Li, Yi-Fan Lu, Xiao-Feng Ding, Yun-Long Pan, Yang Wang and Qing-Yu He**

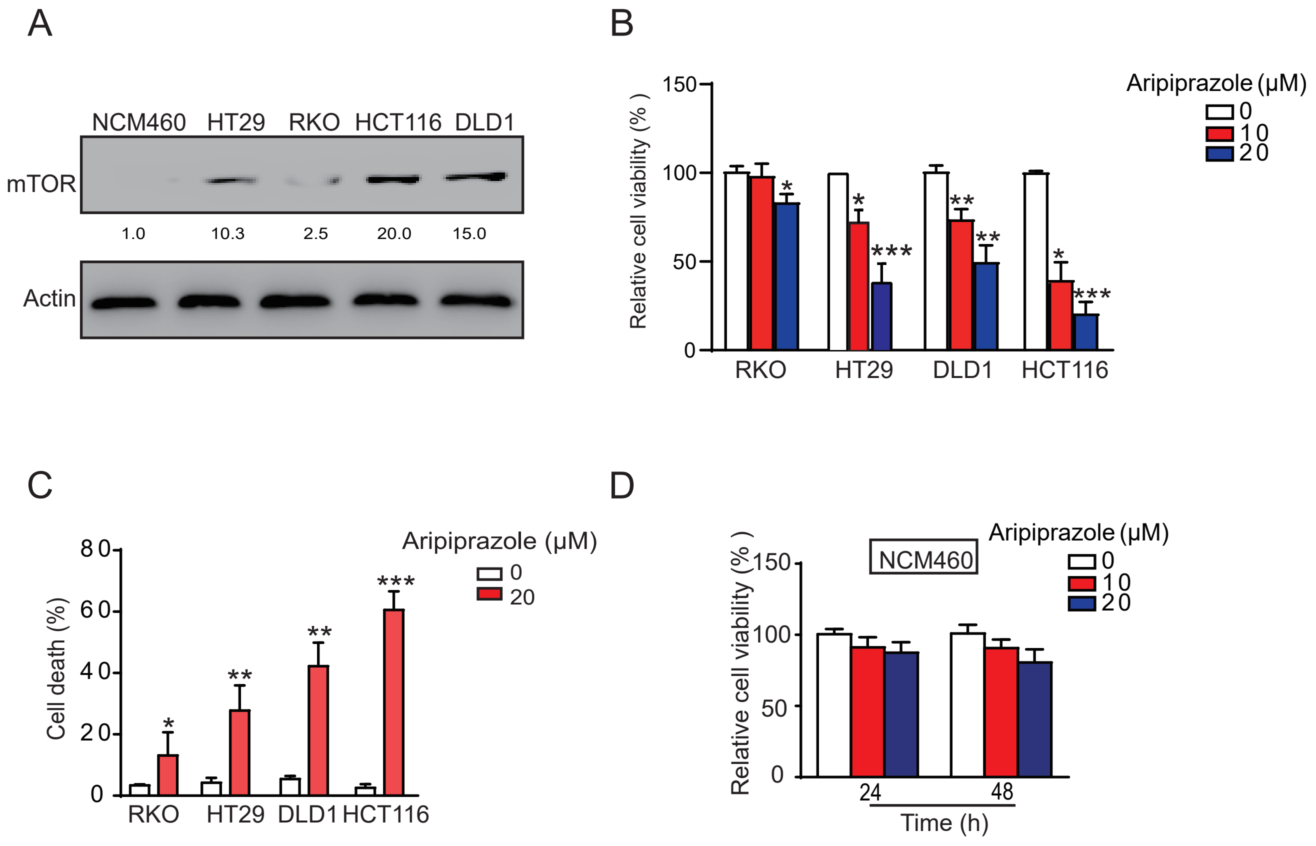

Figure S1

Figure S1. CRC cell with high level of mTOR is sensitive to the aripiprazole. (**A**) The expression of mTOR in the various CRC cells, n = 3/experiments. (**B-C**) WST-1 (**B**) and annexin V-FITC/PI staining assays (**C**) were used to determine the cell viability and cell apoptosis of various CRC cells treated with aripiprazole, n = 3/experiments. (**D**) The cell viability of NCM460 cells treated with aripiprazole was analyzed by WST-1 assay, n = 3/experiments. Bars, SD; **, $P < 0.01$; ***, $P < 0.001$; ns, no significant difference.

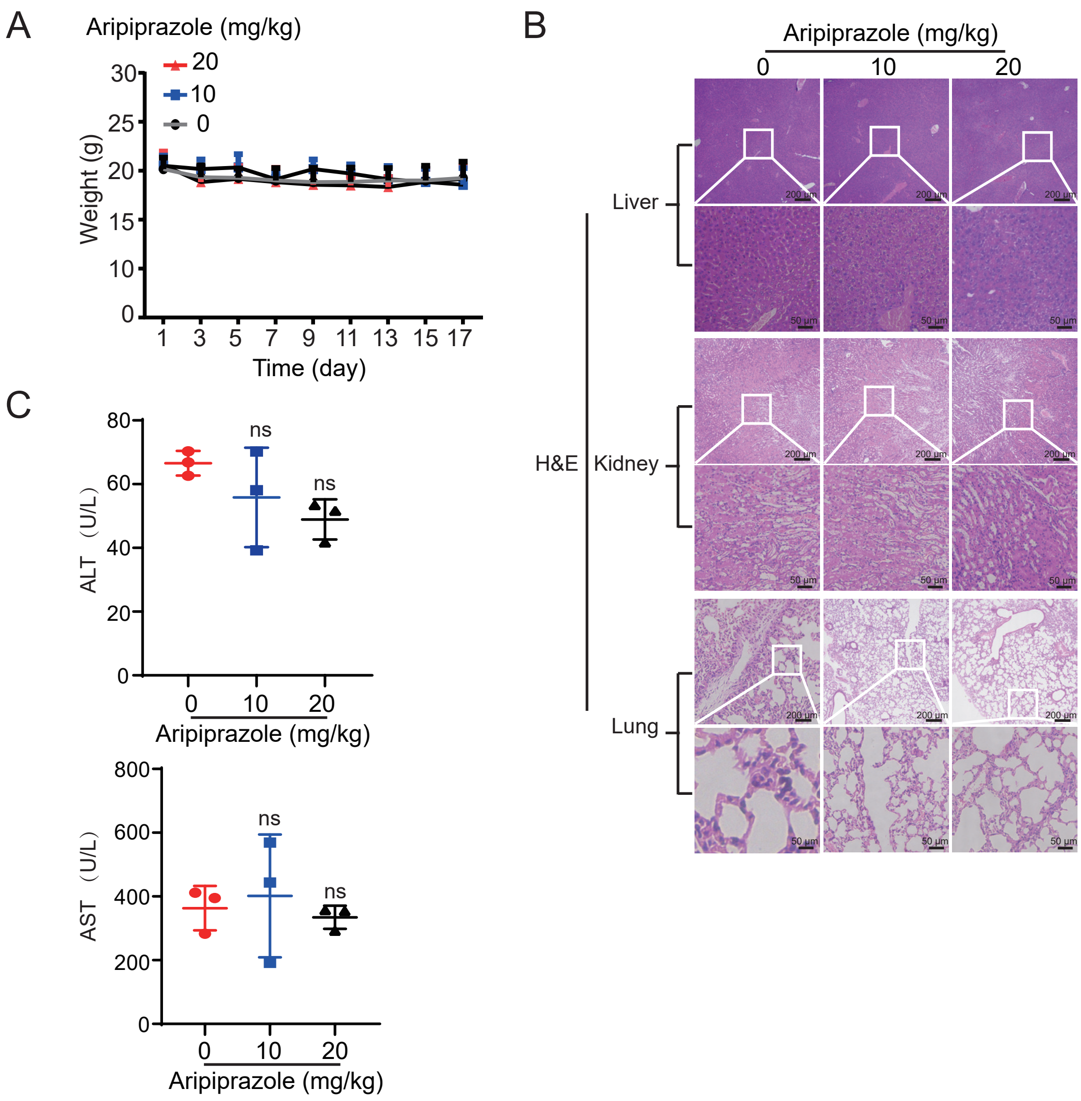

Figure S2

Figure S2. Aripiprazole inhibits the proliferation of CRC cells without causing toxic effect in mice. (**A**) Body weight of nude mice during the experimental period, n = 6 mice/group. (**B**) H&E staining of lung, liver, and kidney collected from mice treated with indicated dosages aripiprazole, n = 3 mice/group. (**C**) Comparison of serum ALT and AST levels between aripiprazole-treated groups and control groups, n = 3 mice/group. Bars, SD; **, $P < 0.01$; ***, $P < 0.001$; ns, no significant difference.

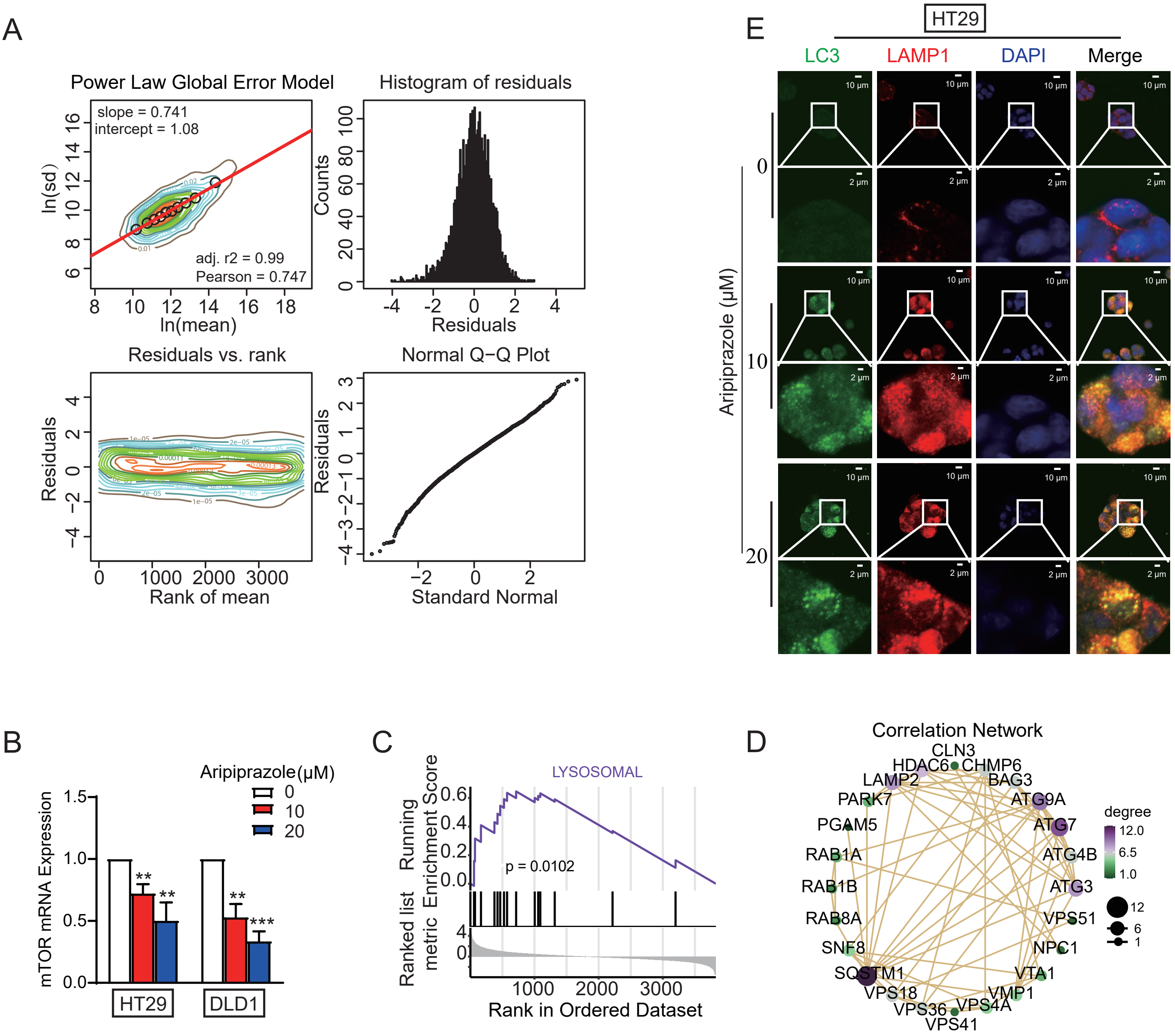

Figure S3

Figure S3. Aripiprazole induces autophagy in CRC cells. (**A**) A PLGEM model was used to fit the abundance of aripiprazole-regulated proteins. (**B**) CRC cells treated with aripiprazole, the mRNA levels of mTOR were determined by RT-qPCR assays, n = 3/experiments. (**C**) GSEA analysis of the aripiprazole-regulated proteins. (**D**) Correlation network of established by aripiprazole-regulated proteins linked to the autophagy. (**E**) Confocal assay of the cellular co-localization of LC3 and LAMP1 in HT29 and DLD1 cells treated with different concentration of aripiprazole (10 μM, 20 μM) or DMSO for 48 h, n = 3/experiments. Bars, SD; **, *P* < 0.01; ***, *P* < 0.001; ns, no significant difference.

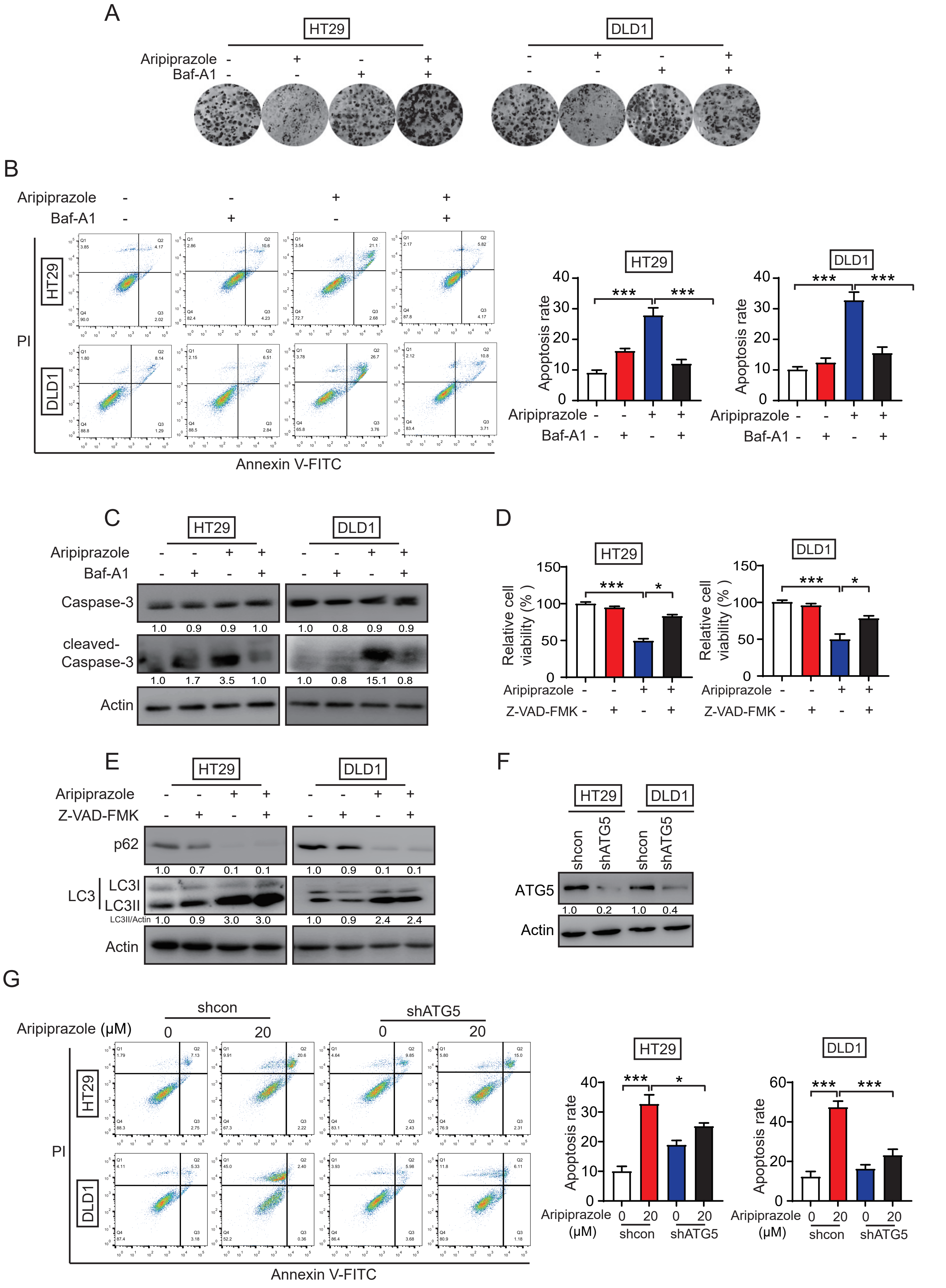
A
HT29
DLD1
Aripiprazole
Baf-A1
B
Aripiprazole
Baf-A1
HT29
DLD1
PI
Annexin V-FITC
Apoptosis rate
C
HT29
DLD1
Aripiprazole
Baf-A1
Caspase-3
1.0 0.9 0.9 1.0
1.0 0.8 0.9 0.9
cleaved-Caspase-3
1.0 1.7 3.5 1.0
1.0 0.8 15.1 0.8
Actin
D
HT29
DLD1
Relative cell viability (%)
Aripiprazole
Z-VAD-FMK
E
HT29
DLD1
Aripiprazole
Z-VAD-FMK
p62
1.0 0.7 0.1 0.1
1.0 0.9 0.1 0.1
LC3
LC3I
LC3II
LC3II/Actin 1.0 0.9 3.0 3.0
1.0 0.9 2.4 2.4
Actin
F
HT29
DLD1
shcon
shATG5
shcon
shATG5
ATG5
1.0 0.2 1.0 0.4
Actin
G
shcon
shATG5
Aripiprazole (μM)
0
20
0
20
HT29
DLD1
PI
Annexin V-FITC
Apoptosis rate
Aripiprazole (μM)

Figure S4

Figure S4. Aripiprazole induces CRC cells apoptosis *via* activating autophagy. (**A**) CRC cells were treated with aripiprazole and/ or pretreated with Baf-A1 (10 nM, 12 h), and then the cell growth was determined by colony-formation assay, n = 3/ experiments. (**B-C**) CRC cells were treated with aripiprazole and / or pretreated with Baf-A1 (10 nM, 12 h), and the cell apoptosis was determined by Annexin V-FITC/PI double staining assay (**B**), and the expression of (cleaved) Caspase 3 was determined by western blot (**C**), n = 3/experiments. (**D-E**) The cell viability and the expressions of p62 and LC3 in the CRC cells exposed to aripiprazole, with or without pretreatment of Z-VAD-FMK (20 μM, 24 h), were compared by WST-1 (**D**) and western blot (**E**), n = 3/experiments. (**F**) ATG5-knockdown CRC cells and control cells were successfully established, n = 3/experiments. **(G)** The CRC cells with or without ATG5 knockdown were treated with aripiprazole (20 μM, 48 h) or DMSO as indicated, and the cell apoptosis was determined by Annexin V-FITC/PI staining assay, n = 3/experiments. Bars, SD; **, $P < 0.01$; ***, $P < 0.001$; ns, no significant difference. n = 3/ experiments.

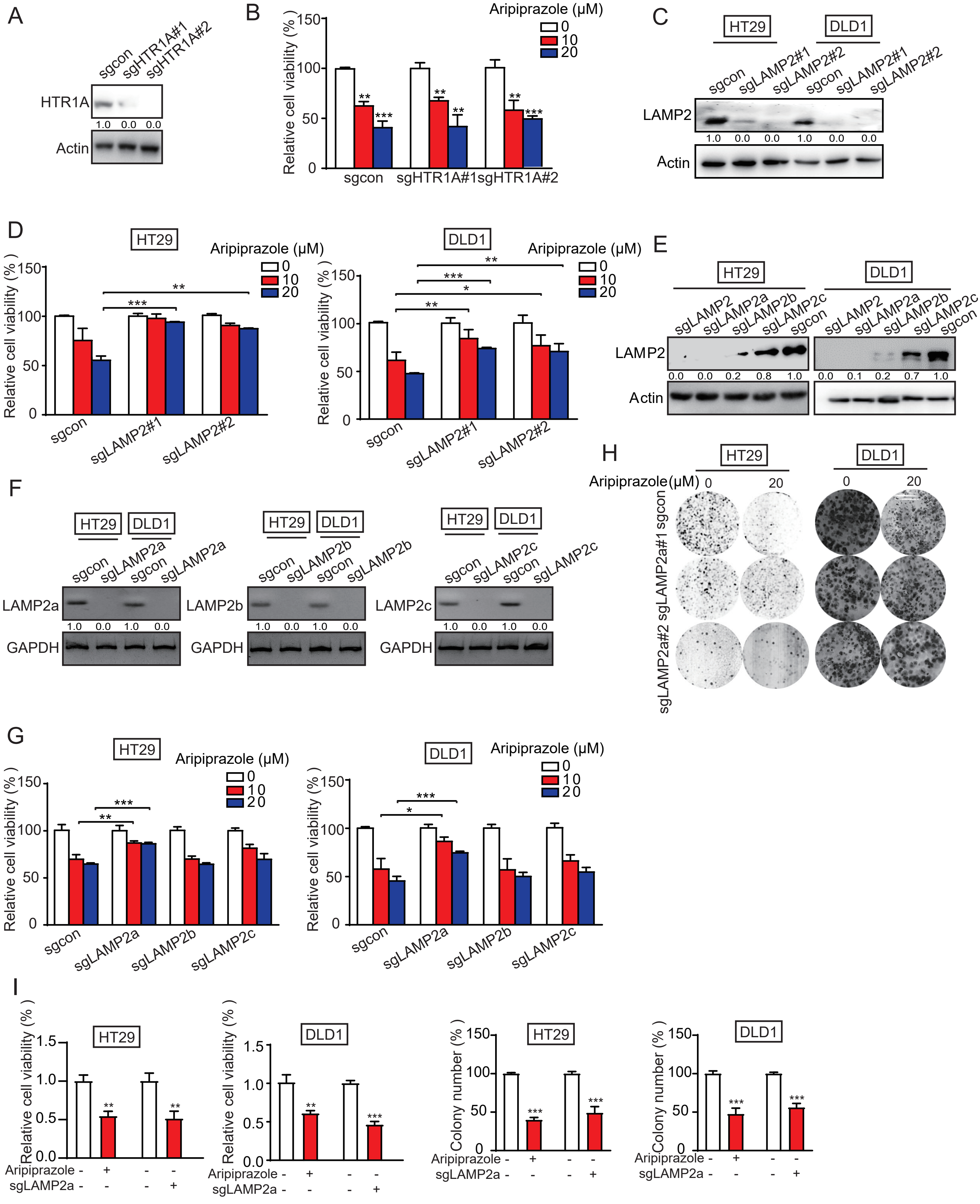

A
sgcon sgHTR1A#1 sgHTR1A#2
HTR1A
1.0 0.0 0.0
Actin
B
Aripiprazole (μM)
0
10
20
Relative cell viability (%)
sgcon sgHTR1A#1 sgHTR1A#2
C
HT29
DLD1
sgcon sgLAMP2#1 sgLAMP2#2 sgcon sgLAMP2#1 sgLAMP2#2
LAMP2
1.0 0.0 0.0 1.0 0.0 0.0
Actin
D
HT29
DLD1
Aripiprazole (μM)
Relative cell viability (%)
sgcon sgLAMP2#1 sgLAMP2#2
E
sgLAMP2 sgLAMP2a sgLAMP2b sgLAMP2c sgcon
LAMP2
0.0 0.0 0.2 0.8 1.0
0.0 0.1 0.2 0.7 1.0
Actin
F
sgcon sgLAMP2a sgcon sgLAMP2a
LAMP2a
LAMP2b
LAMP2c
1.0 0.0 1.0 0.0
GAPDH
G
sgcon sgLAMP2a sgLAMP2b sgLAMP2c
H
Aripiprazole(μM) 0 20
sgcon
sgLAMP2a#1
sgLAMP2a#2
I
Relative cell viability (%)
Colony number (%)
Aripiprazole - + - -
sgLAMP2a - - - +

Figure S5

Figure S5. LAMP2a, but not HTR1A, mediates the anticancer effect of aripiprazole in CRC. (**A-B**) HTR1A-deficient CRC cells and control cells were successfully established (**A**), then treated with aripiprazole (10 μM, 20 μM) or DMSO, and then the cell viability was determined by WST-1 assay (**B**), n = 3/experiments. (**C-D**) Both CRC cells with or without LAMP2-deficience (**C**) were treated with aripiprazole (10 μM, 20 μM) or DMSO, the cell viability was determined by WST-1 assay (**D**), n = 3/experiments. (**E-F**) The knockout effect of LAMP2a, LAMP2b and LAMP2c in HT29 and DLD1 cells was determined by western blot and qRT-PCR assays, n = 3/experiments. (**G**) The cell viability of LAMP2a-deficient, LAMP2b-deficient, LAMP2c-deficient and control CRC cells treated with aripiprazole (10 μM, 20 μM, 48 h) or DMSO was determined by WST-1 assay, n = 3/experiments. (**H**) The colony formation assay of LAMP2a-deficient and control CRC cells with indicated treatment, n = 3/experiments. Bars, SD; **, $P < 0.01$; ***, $P < 0.001$; ns, no significant difference.

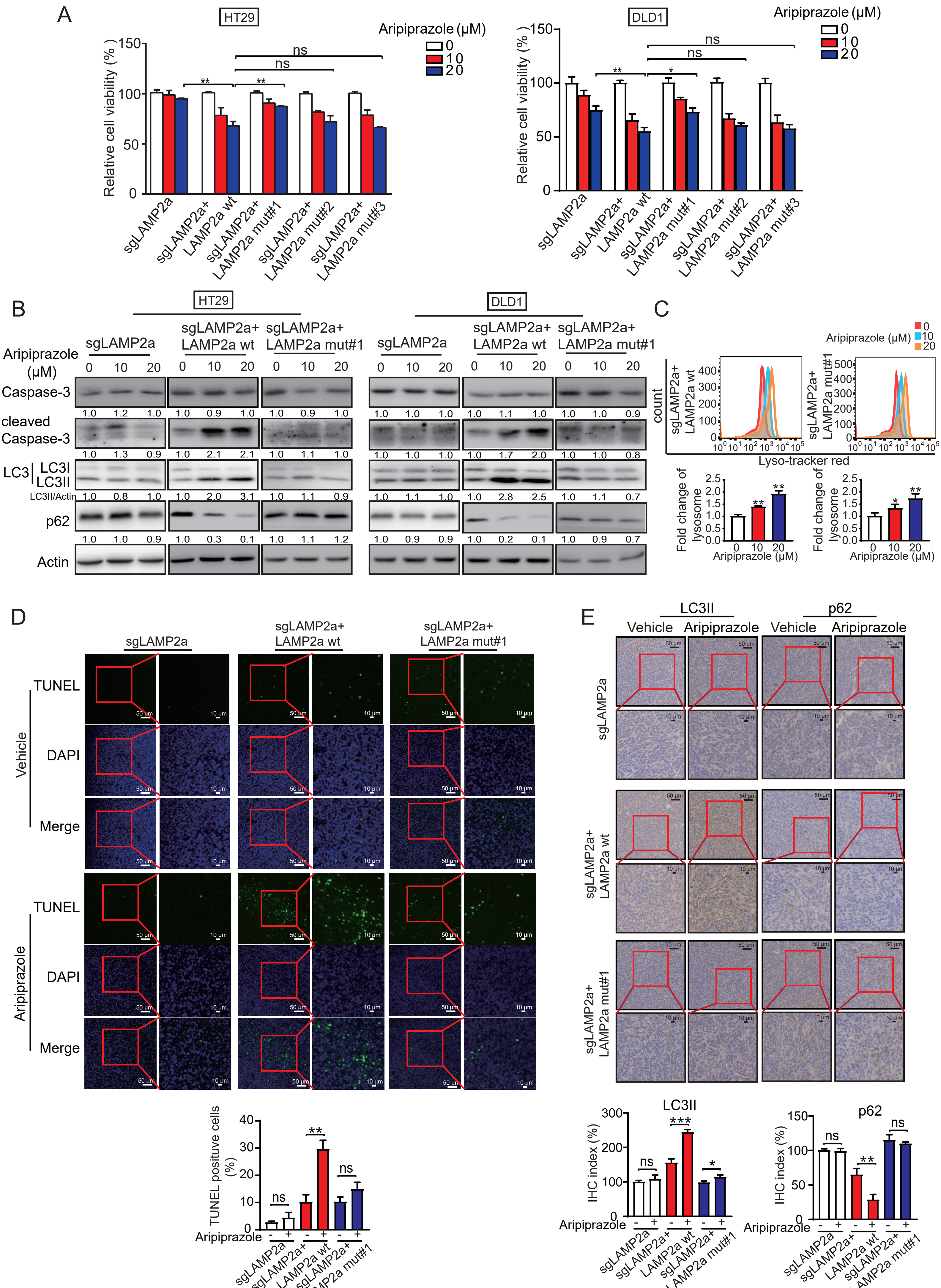

A
HT29
DLD1
Aripiprazole (μM)
0
10
20
Relative cell viability (%)
ns
**
*
sgLAMP2a
sgLAMP2a+ LAMP2a wt
sgLAMP2a+ LAMP2a mut#1
sgLAMP2a+ LAMP2a mut#2
sgLAMP2a+ LAMP2a mut#3
B
Caspase-3
cleaved Caspase-3
LC3 LC3I LC3II
LC3II/Actin
p62
Actin
C
count
Lyso-tracker red
Fold change of lysosome
D
TUNEL
DAPI
Merge
Vehicle
Aripiprazole
TUNEL positive cells (%)
E
LC3II
p62
IHC index (%)

Figure S6

Figure S6. Lys-401and three-His site (H402-404) of LAMP2a are required for anticancer activity of aripiprazole. (**A**) WST-1 assay was used to determine the cell viability of indicated CRC cells treated with aripiprazole (0-20 μM) for 48 h, n = 3/experiments. (**B**) Western blots of Caspase3, cleaved-Caspase3, LC3 and p62 in indicated LAMP2a-deficient cells and with re-overexpression of WT or mutated LAMP2a treated with aripiprazole (0-20 μM) for 48 h, n = 3/experiments. (**C**) The lysosomes content of LAMP2a-deficient cells with re-overexpression of WT or mutated LAMP2a mut#1 treated with aripiprazole (0-20 μM) for 48 h, n = 3/ experiments. (**D-E**) The apoptosis of tumor xenografts was analyzed by TUNEL assay (**D**) and the autophagy of tumor xenografts was stained by IHC of LC3II and p62 index (**E**), n = 3 mice/group. Bars, SD; **, P < 0.01; ***, P < 0.001; ns, no significant difference.

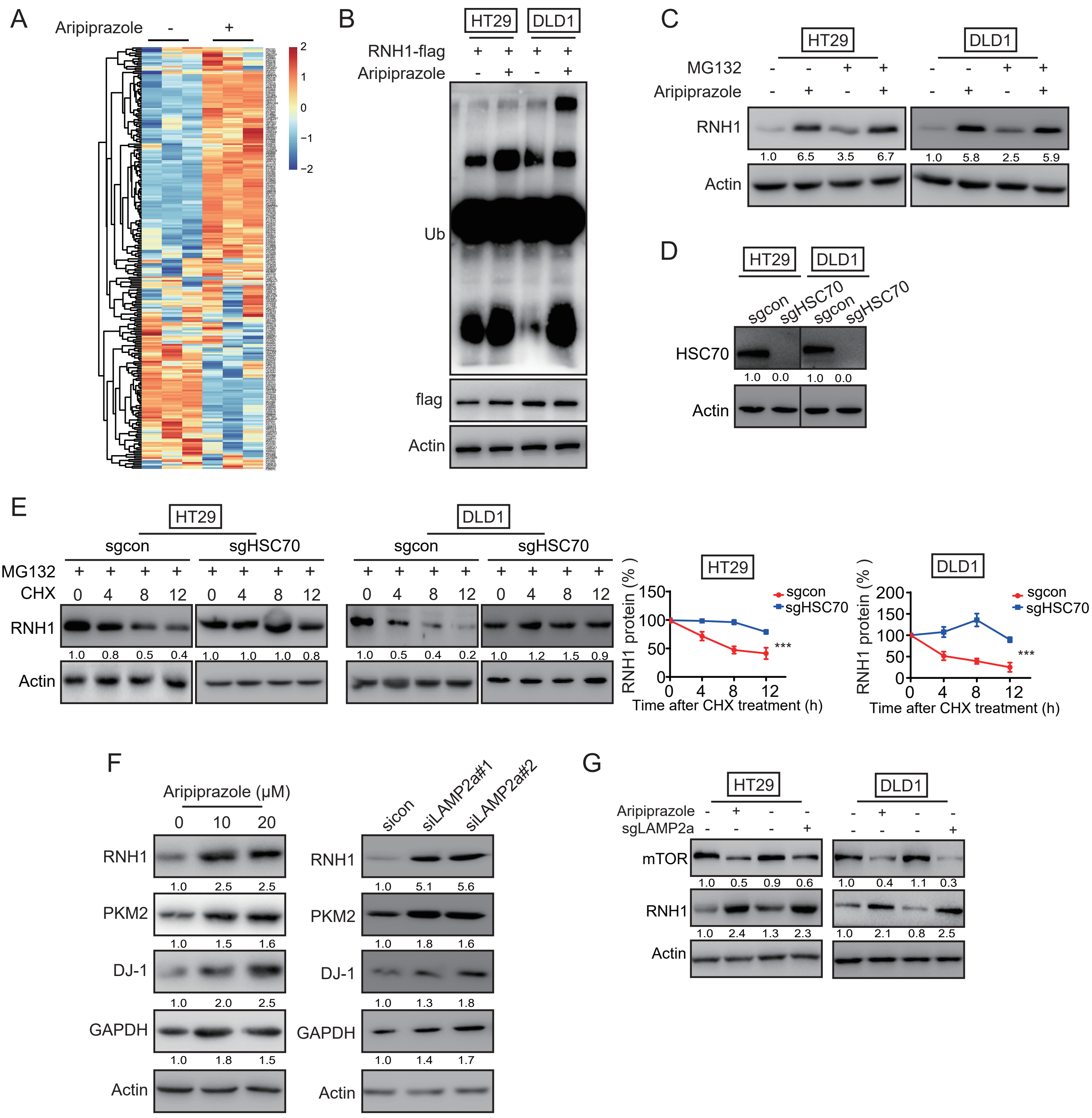

A
Aripiprazole - +
B
HT29 DLD1
RNH1-flag + + + +
Aripiprazole - + - +
Ub
flag
Actin
C
HT29 DLD1
MG132 - - + + - - + +
Aripiprazole - + - + - + - +
RNH1
1.0 6.5 3.5 6.7 1.0 5.8 2.5 5.9
Actin
D
HT29 DLD1
sgcon sgHSC70 sgcon sgHSC70
HSC70
1.0 0.0 1.0 0.0
Actin
E
HT29 DLD1
sgcon sgHSC70 sgcon sgHSC70
MG132
CHX 0 4 8 12 0 4 8 12 0 4 8 12 0 4 8 12
RNH1
1.0 0.8 0.5 0.4 1.0 1.0 1.0 0.8 1.0 0.5 0.4 0.2 1.0 1.2 1.5 0.9
Actin
HT29
sgcon
sgHSC70
RNH1 protein (%)
Time after CHX treatment (h)
***
DLD1
sgcon
sgHSC70
RNH1 protein (%)
Time after CHX treatment (h)
***
F
Aripiprazole (μM)
0 10 20
RNH1
1.0 2.5 2.5
PKM2
1.0 1.5 1.6
DJ-1
1.0 2.0 2.5
GAPDH
1.0 1.8 1.5
Actin
sicon siLAMP2a#1 siLAMP2a#2
RNH1
1.0 5.1 5.6
PKM2
1.0 1.8 1.6
DJ-1
1.0 1.3 1.8
GAPDH
1.0 1.4 1.7
Actin
G
HT29 DLD1
Aripiprazole - + - - - + - -
sgLAMP2a - - - + - - - +
mTOR
1.0 0.5 0.9 0.6 1.0 0.4 1.1 0.3
RNH1
1.0 2.4 1.3 2.3 1.0 2.1 0.8 2.5
Actin

Figure S7

Figure S7. Aripiprazole induces the degradation of RNH1 in a lysosome-dependent manner. (**A**) Analysis of binding proteins that contain KFERQ-like motif of LAMP2a, n = 3/experiments. (**B**) Immunoprecipitation was performed in CRC cells transfected with RNH1-flag plasmids and treated with DMSO or Aripiprazole, and ubiquitin of RNH1 was detected, n = 3/experiments. (**C**) Western blot was performed in CRC cells with the indicated treatment, and RNH1 expression was detected, n = 3/experiments. (**D**) HSC70-deficient CRC cells and control cells were successfully established, n = 3/ experiments. (**E**) The effect of HSC70 on RNH1 protein stability was examined by using a CHX (100 μg/mL) chase assay for indicated periods of time, n = 3/ experiments. (**F**) The expressions of CMA substrates in CRC cells were determined by western blot, n = 3/experiments. (**G**) The expression of mTOR and RNH1 in LAMP2a-deficient and control CRC cells treated with aripiprazole (10 μM for HT29; 20 μM for DLD1, 48 h) was determined by western blot, n = 3/experiments. Bars, SD; **, $P < 0.01$; ***, $P < 0.001$; ns, no significant difference. n = 3/experiments.

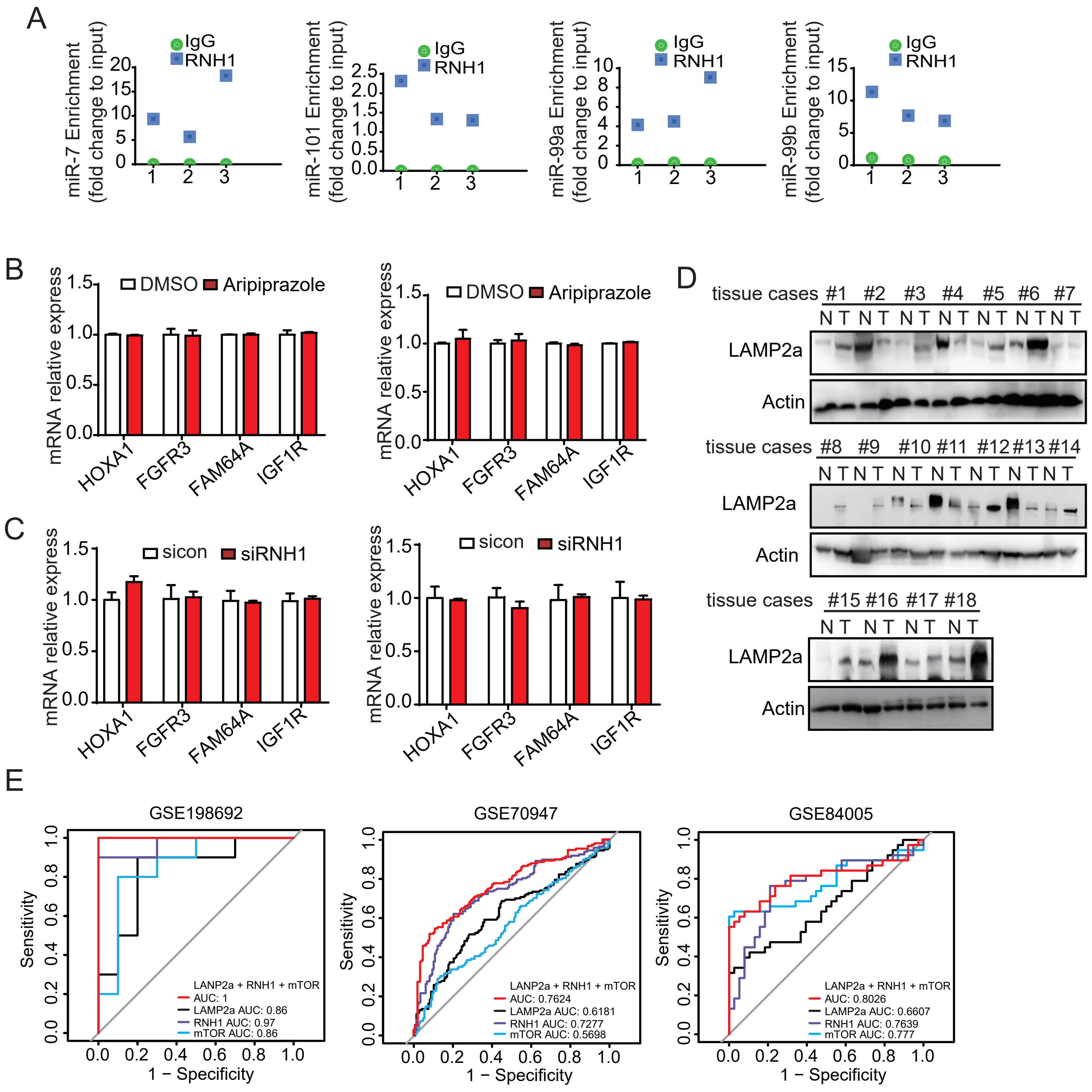

A
IgG
RNH1
miR-7 Enrichment (fold change to input)
miR-101 Enrichment (fold change to input)
miR-99a Enrichment (fold change to input)
miR-99b Enrichment (fold change to input)
B
DMSO
Aripiprazole
mRNA relative express
HOXA1
FGFR3
FAM64A
IGF1R
C
sicon
siRNH1
D
tissue cases #1 #2 #3 #4 #5 #6 #7
tissue cases #8 #9 #10 #11 #12 #13 #14
tissue cases #15 #16 #17 #18
N T
LAMP2a
Actin
E
GSE198692
GSE70947
GSE84005
Sensitivity
1 − Specificity
LANP2a + RNH1 + mTOR
AUC: 1
LAMP2a AUC: 0.86
RNH1 AUC: 0.97
mTOR AUC: 0.86
AUC: 0.7624
LAMP2a AUC: 0.6181
RNH1 AUC: 0.7277
mTOR AUC: 0.5698
AUC: 0.8026
LAMP2a AUC: 0.6607
RNH1 AUC: 0.7639
mTOR AUC: 0.777

Figure S8

Figure S8. RNH1 bind to mTOR mRNA and miR-99a. (**A**) The enrichment of RNH1 to mTOR mRNA and miRNAs was detected by RIP assay. Results were shown as individual enrichment for each independent experiment, n = 3/experiments. (**B-C**) qRT-PCR assay was used to determine mRNA level of HOXA1, FGFR3, FAM64A and IGF1R in CRC cells treated with aripiprazole (20 μM, 48 h) or silencing RNH1, n = 3/experiments. (**D**) The majority of tumor cases had a stronger expression of LAMP2a in tumors (T) than in adjacent normal tissues (N). (**E**) ROC curve analysis of CRC (GSE198692, GSE70947, GSE84005). Bars, SD; **, $P < 0.01$; ***, $P < 0.001$; ns, no significant difference.

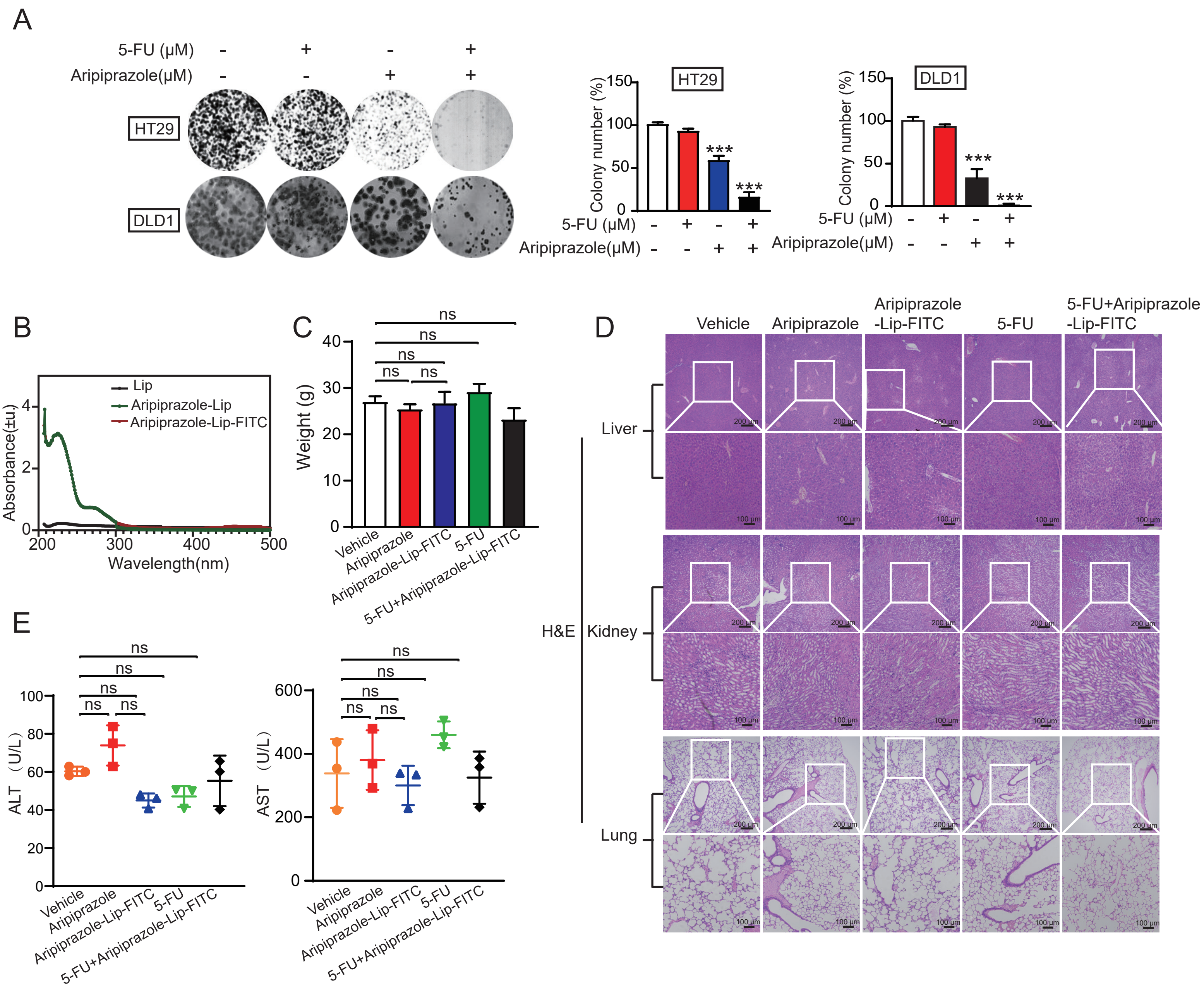

Figure S9

Figure S9. Combined treatment of aripiprazole and 5-FU without observed toxic effect in mice. (**A**) The colony formation ability of the CRC cells treated with 5-FU (10 μM), aripiprazole (10 μM) alone, or the combination of 5-FU and aripiprazole, n = 3/experiments. (**B**) UV spectrum, n = 3/experiments. (**C**) Body weight of nude mice during the experimental period, n = 6 mice/group. (**D**) H&E staining of lung, liver, and kidney collected from mice with indicated treatment, n = 3 mice/group. (**E**) Comparison of serum ALT and AST levels in mice with indicated treatment, n = 3 mice/group. Bars, SD; **, $P < 0.01$; ***, $P < 0.001$; ns, no significant difference.
