# Supplementary material for: The Antipsychotic Drug Aripiprazole Suppresses Colorectal Cancer by Targeting LAMP2a to Induce RNH1/miR‐99a/mTOR‐Mediated Autophagy and Apoptosis
Source: Adv Sci (Weinh). 2024 Nov 8;11(48):2409498. doi: 10.1002/advs.202409498 (PMC11672294; doi:10.1002/advs.202409498)
Supplement: Supplementary file 2 — Supplemental Table 1 [file ADVS-11-2409498-s002.docx]

**The sequences of primers for cloning and mutation**

**pLenti CMV GFP Hygro**

| LAMP2a-wt-up | GACACCGACTCTAGAGGATCCATGGTGTGCTTCCGCCTCT |
| --- | --- |
| LAMP2a-wt-dw | ATGGTGGCGACCGGTGGATCCCTAAAATTGCTCATATCCAGCATGA |
| LAMP2a-mut2-up | GCTGGCTGCTTTTATTGGTCTCAAGCACCATCA |
| LAMP2a-mut2-dw | CAATAAAAGCAGCCAGCAACACTAGAATAAGTACTCC |
| LAMP2a-mut3-up | GGCTTATTTTGCTGGTCTCAAGCACCATCATGCTG |
| LAMP2a-mut3-dw | GACCAGCAAAATAAGCCAGCAACACTAGAATAAG |
| LAMP2a-mut1-up | CTCGCGGCCGCTGCTGCTGGATATGAGCAATTTTAGGG |
| LAMP2a-mut1-dw | AGCAGCAGCGGCCGCGAGACCAATAAAATAAGCCAGCAA |

**pET-28b**

| His- LAMP2a-wt-up | TTGTCGACGGAGCTCGAATTCATGGTGTGCTTCCGCCTCT |
| --- | --- |
| His- LAMP2a-wt-dw | AATGGGTCGGGATCCGAATTCCTAAAATTGCTCATATCCAGCATGA |
| His- LAMP2a-mut1-up | CTCCGCGGCCGCTGTGCTGGATATGAGCAATTTTAGGAA |
| His-LAMP2a-mut1-dw | AGCACAGCGGCCGCGGAGACCAATAAAATAAGCCAGCAA |

**pcDNA3.1**

| Flag-RNH1-Q147A-up | AAAAGCTGGCGCTGGAGTATTGCAGCCTCTCGG |
| --- | --- |
| Flag-RNH1-Q147A -dw | ACTCCAGCGCCAGCTTTTCCAGGCGGCACTGGG |

**The sequences of sgRNA**

| sgLAMP2#1 | CACCGC ATGATGTTGTCCAACACTAC |
| --- | --- |
| sgLAMP2#2 | CACCGC CAAGAACATCCCAGTAGTGT |
| sgLAMP2a#1 | ACUUCCUAACACGCAUAUUU |
| sgLAMP2a#2 | AGTACTTATTCTAGTGTTGC-3 |
| sgLAMP2b | CAACUUCAAGUAACUAAGAC |
| sgLAMP2c | GCAAGCGCAAUUCUCUAUUU |

**The sequences of siRNA**

| siRNH1#1 | TACGACATTTACTGGTCTGAGGAGATGGA |
| --- | --- |
| siRNH1#2 | TGCTCTGGTTGGCCGACTGCGATGTGAGT |
| siAgo2#1 | AACTTCTGGGCTGTTCTCGCT |
| siAgo2#2 | AAGGATATGCCTTCAAGCCTC |

**The sequences of primers for RT-qPCR**

| MTMR3-up | AGCAGAGTGGGCTCAGTGTT |
| --- | --- |
| MTMR3-dw | ACTGTCCACGTTTGGTCCTC |
| HOXA1-up | AGTTGGAGAGTACGGCTACCTG |
| HOXA1-dw | TGCAGGGATGCAGCGATCTCCAC |
| IGF1R-up | AACCCCAAGACTGAGGTGTG |
| IGF1R-dw | TGACATCTCTCCGCTTCCTT |
| FAM64A-up | TTCTCGGTGGCAGAACATGG |
| FAM64A-dw | GTCTCCTGATGGCTGACCAC |
| CDC25A-up | GAGGAGTCTCCACCTGGAAGTACA |
| CDC25A-dw | GCCATTCAAAACAGATGCCATAA |
| FGFR3-up | CGTACTGTGCCACTTCAGTG |
| FGFR3-dw | CCAGCAGCTTCTTGTCCATC |
| mTOR-up | AGAAACTGCACGTCAGCACCA |
| mTOR-dw | CCATTCCAGCCAGTCATCTTTG |
| GAPDH-up | CTCTGCTCCTCCTGTTCGAC |
| GAPDH-dw | GCGCCCAATACGACCAAATC |
| miR-7 | TGGAAGACTAGTGATTTTGTTG |
| miR-101 | GTACAGTACTGTGATAACTGA |
| miR-193a-5p | TGGGTCTTTGCGGGCGAGATGA |
| miR-199a-3p | ACAGTAGTCTGCACATTGGTTA |
| miR-96-5p | TTTGGCACTAGCACATTTTTGCT |
| miR-99a | AACCCGTAGATCCGATCTTGTG |
| miR-99b | CACCCGTAGAACCGACCTTGCG |
| U6 | CTCGCTTCGGCAGCACA |
| LAMP2a-up | GTGCAACAAAGAGCAGACTGT |
| LAMP2a-dw | GGCACAAGGAAGTTGTCGTC |
| LAMP2b-up | AGAGTGTTCGCTGGATGATG |
| LAMP2b-dw | TGCCAATTACGTAAGCAATCA |
| LAMP2c-up | AAGGGTTCAGCCTTTCAATG |
| LAMP2c-dw | ACAATTATAAGGAAGCCCAAGG |
